# Multifunctional nanosponges in cancer therapy: Integrating targeted drug delivery and theranostic potential

**DOI:** 10.1016/j.ijpx.2025.100421

**Published:** 2025-10-20

**Authors:** Sandesh Ramchandra Jadhav, Ashutosh Gupta, Viola Colaco, Moumita Saha, Amatha Sreedevi, Deepanjan Datta, Sudheer Moorkoth, Virendra S. Ligade, Namdev Dhas

**Affiliations:** a Department of Pharmaceutics, Manipal College of Pharmaceutical Sciences, Manipal Academy of Higher Education, Manipal 576104, Karnataka, India; b Department of Pharmaceutical Quality Assurance, Manipal College of Pharmaceutical Sciences, Manipal Academy of Higher Education, Manipal 576104, Karnataka, India; c Department of Pharmaceutical Regulatory Affairs, Manipal College of Pharmaceutical Sciences, Manipal Academy of Higher Education, Manipal 576104, Karnataka, India

**Keywords:** Cancer therapy, Nanosponges, Photothermal therapy, Photodynamic therapy, Targeted drug delivery, Pharmacokinetics

## Abstract

Cancer remains a leading cause of mortality, with conventional therapies often limited by systemic toxicity, poor drug bioavailability, and the emergence of drug resistance. Multifunctional nanosponges represent an innovative nanotherapeutic platform for cancer management, integrating targeted drug delivery and theranostic functionalities to overcome limitations of conventional therapies. These nanosponges exhibit high encapsulation efficiency for hydrophilic and hydrophobic therapeutics and biologics. Surface functionalization with ligands enables selective tumor targeting *via* receptor-mediated interactions and enhanced permeability and retention (EPR) effect for preferential drug accumulation in cancer tissues. Stimuli-responsive nanosponges, endogenous and exogenous stimuli, facilitate controlled drug release within the tumor microenvironment, minimizing systemic toxicity. Theranostically, nanosponges incorporate imaging moieties for real-time visualization *via* MRI, CT, or fluorescence imaging, enabling concurrent diagnostics and therapy. Advanced designs, such as RBC-membrane-coated or DNAzyme-based nanosponges, co-deliver chemotherapeutic agents and gene-silencing constructs, achieving synergistic effects through combinational therapies. Nanosponges offer tunable physicochemical properties and multifunctionality, positioning them as a transformative tool for precision oncology. Collectively, these advances establish multifunctional nanosponges as a versatile and clinically translatable platform, potentially overcoming current therapeutic barriers and redefining strategies for precision cancer management.

## Graphical abstract

**Figure:**
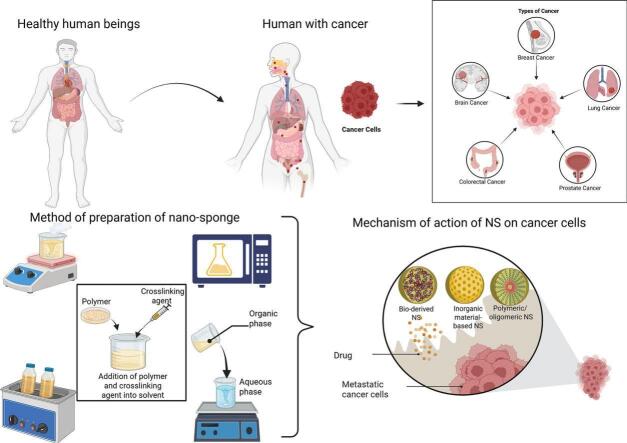

## 1 Introduction

Cancer has become one of the leading causes of death worldwide, due to various factors, including challenges in accurately diagnosing the disease and difficulties in finding effective, affordable treatments (Hindorff et al., 2011). According to an NCI study, cancer is currently a significant social, economic, and scientific issue since estimates suggest that by 2030, there will be 20 million new instances of the disease worldwide, and 13 million more people will have died from it. Therefore, this will be a terrifying barrier for humanity to overcome and confront such a grave problem. To enhance the prevention, diagnosis, and treatment of many malignancies and guarantee that survivors lead longer, higher-quality lives, cancer research is essential. Additionally, research aids in determining the origin of cancer and is paving the way for better diagnostic and therapeutic approaches (Al-Ostoot and Khanum, 2024). This review has discussed an overview of cancer, its biology, etiology, recent cancer therapies, and modified drug delivery systems using nanosponges (NSs) for theranostic purposes.

Cancer is a general word for a set of complicated disorders characterized by the unrestricted growth, proliferation, and invasion of any surrounding tissues by cancerous cells (Gupta et al., 2025b; Hindorff et al., 2011). This can also explain a sickness that develops when aberrant development and division of cancer cells allow them to infiltrate and destroy natural, healthy organs anywhere in the body (Yang et al., 2020). Through further intricate processes, these aberrant cells, also known as cancer cells, tumor cells, or malignant cells, can infect healthy organs within the body (De Visser and Joyce, 2023). The majority of malignancies and abnormal cells that make up malignant tissue are often identified as being formed by cancerous cells that can spread to other regions of the body, such as breast, colorectal, and lung cancer (Feng et al., 2018a, Feng et al., 2018b). Cancer will remain a significant global health concern, particularly in developing nations, where innovative therapeutic approaches are being tested (Petersen, 2009). Until now, several techniques, including radiation, chemotherapy, immunotherapy, gene therapy, and surgery, have been proposed and employed to treat malignant tissues, particularly metastases (Abad et al., 2021; Gupta et al., 2025c; Naik et al., 2025).

It is important to note that while not all tumors are cancerous, all malignant tumors exhibit aggressive behaviour. Non-cancerous tumors are classified as benign, whereas cancerous tumors are termed malignant. Many malignancies grow into solid tumors that can be either benign or malignant and become solid masses of tissue, free of fluids or cysts (McCarville et al., 2006). On the other hand, blood malignancies, such as leukaemia and lymphoma, typically affect the bone marrow, lymphatic system, and blood cells rather than forming a solid tumor. Additionally, when these tumors grow, some cancer cells may separate and move to different body areas *via* the circulatory or lymphatic systems, creating secondary tumors far from the initial tumor site. In contrast to malignant tumors, benign tumors stay contained and do not spread to other parts of the body or invade nearby tissues (Anderson and Simon, 2020).

**Fig. 1:**
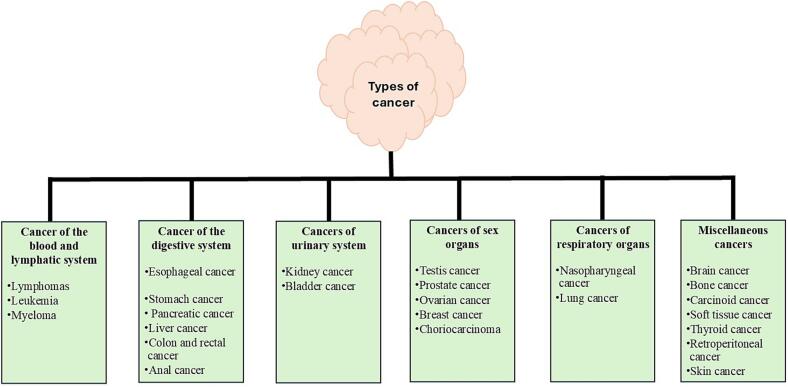
Pictorial representation of types of cancer.

Certain primary cancer forms, including sarcoma, myeloma, lymphoma, leukaemia, and carcinoma, are also referred to by several distinct clinical terms from a histological perspective (Paul et al., 2019). The difference between them is where the cancer first started before spreading to another area of the surrounding tissues. Furthermore, when malignant cells enter the circulation, they spread to many of the tissues linked to metastasis creation in addition to the original organs where the cancer first appeared (Keskinov and Shurin, 2015). For many reasons, including the difficulties in accurately diagnosing the disease and the difficulty in locating particular, cost-effective target therapies, cancer has emerged as a leading cause of mortality worldwide (Mattiuzzi and Lippi, 2019). This high fatality rate is caused in part by the lack of effective therapeutic strategies that target cancer cells specifically rather than other cells. Radiation and chemotherapy are two well-known and often used cancer treatment modalities. Both have serious adverse effects that, in more advanced instances, might be fatal. One of the main disadvantages of these therapies is that they destroy healthy tissues or cells directly, which can harm all tissues in the body in the majority of advanced cancer stages. This review mainly focuses on the targeted delivery of medicaments for various cancers *via* NSs for diagnosis and treatment. The genesis of cancer is a sequence of events that, through intermediary stages, harm the genetic material of the original cells, eventually resulting in unchecked cell proliferation, tumor formation, and finally carcinogenic tissue. Anything that raises the probability of getting cancer is considered a risk factor. Most cancer types are linked to exposure to various variables, such as behavioral, environmental, and lifestyle factors (Zanini et al., 2021). In this regard, exposure to intense sunlight is a risk factor that is likely to cause skin cancer. There are two categories of cancer risk factors: those that can be changed, like smoking, drinking alcohol, and being overweight, and those that cannot, such as gender, inherited genetic abnormality, and age. Additionally, many variables have been shown in studies to play a significant role in either raising the risk of acquiring cancer or promoting its growth and spread (Das et al., 2020). Tobacco use (Brown et al., 2018), excessive alcohol use, nutrition and physical activity, viruses and other diseases (Anand et al., 2008), age and cancer risk (Risk Factors: Age - NCI [WWW Document], 2015), and radiation from the sun as well are the key risk factors that may cause cancer (Downs et al., 2020). Cancer may be classified in different ways. One of the ways is according to histology, classified into mainly six groups, including: sarcoma, myeloma, leukaemia, lymphoma, blastoma, carcinoma, and mixed type (Al-Ostoot and Khanum, 2024). Based on the organ affected, cancer is classified into many types, as shown in the Fig. 1 (Koul, 2019).

Nanosponges are nanoscale, porous, three-dimensional structures formed by crosslinking, designed to encapsulate and deliver a variety of therapeutic and diagnostic agents in a controlled, sustained, or targeted manner. Depending on their composition, NSs can be classified as polymeric, inorganic, or biomimetic, each offering unique advantages for cancer therapy (Sharma et al., 2023). These NSs have been investigated for the treatment of various malignancies, including breast (Shah et al., 2023), lung (Deinavizadeh et al., 2023), prostate (Minelli et al., 2012), brain (Yin et al., 2024), and colorectal (Mohite et al., 2024). NSs are an advanced drug delivery system that can encapsulate both hydrophobic and hydrophilic medicaments, enhancing their solubility, stability, and bioavailability. NSs provide targeted drug release through surface modifications to reduce systemic toxicity or, combined with diagnostic and imaging agents, extend their application to theranostic approaches. NSs also give sustained release and can carry a wide variety of molecules such as proteins, enzymes, and gases, making them suitable for different modes of administration such as oral, topical, and parenteral. These diverse and concurrent capabilities make NSs multifunctional carriers in modern therapy and diagnosis (Barik et al., 2025; Gandhi et al., 2025; Shruti I Meshram et al., 2024; Subramanian, 2017).

## 2 Pathophysiology of cancer

Genetic alterations that interfere with the regular control of cell division and death are the root cause of cancer. These mutations usually impact three gene types: DNA repair genes, tumor suppressor genes, and proto-oncogenes. When proto-oncogenes like RAS and MUC are transformed into oncogenes, they increase growth signals and encourage uncontrollable, rapid cell division (Dakal et al., 2024). For example, RAS mutations, which are found in around 30 % of all malignancies (including colorectal and pancreatic), trigger the MAPK/ERK pathway, which promotes growth even in the absence of outside stimuli. As cellular brakes, tumor suppressor genes such as TP53 and RB stop the cell cycle at checkpoints (such as G1/S) to repair damaged DNA or trigger apoptosis if the damage is irreversible (Borrero and El-Deiry, 2021). This defence system is eliminated by TP53 mutations, which are present in 50 % of malignancies and permit damaged cells to multiply. Abnormalities in DNA repair genes, such as BRCA1/2, prevalent in breast and ovarian malignancies, increase genomic instability. BRCA1 mutations raise the lifetime risk of breast cancer to 70 %. Environmental carcinogens such as tobacco smoke, UV rays, or oncogenic viruses (like HPV in cervical cancer) frequently cause these genetic changes by introducing mutations through DNA adducts or double-strand breaks (Wang et al., 2024a, Wang et al., 2024b).

Epigenetic alterations, which affect gene expression without impacting the DNA sequence, also play a role in cancer. When tumor suppressor gene promoters, like PTEN in endometrial cancer, are hypermethylated, their expression is silenced, simulating genetic loss. In liver cancer, global hypomethylation upregulates MYC, activating oncogenes by de-repressing their transcription. Inhibiting cell cycle progression, histone changes such as deacetylation by HDACs compress chromatin and reduce tumor suppressor genes, including CDKN1A (p21) (Abdul et al., 2017; Vasilatou et al., 2013). While folate deficiency reduces methyl group availability, upsetting epigenetic control, environmental variables, such as prolonged exposure to cigarette smoke, promote DNA methylation in lung cancer. This demonstrates how epigenetic modifications worsen genetic flaws. In *H. pylori*-induced gastric cancer, hypermethylation of mismatch repair genes such as MLH1 causes microsatellite instability, a prelude to cancer (Matsusaka et al., 2014).

A key factor in the development of cancer is the tumor microenvironment (TME), which fosters the growth of tumors. A pro-tumorigenic environment is facilitated by chronic inflammation, which is frequently brought on by infections such as *H. pylori* in stomach cancer (Biray Avci et al., 2024). Released during inflammation, cytokines including TNF-α, IL-1β, and IL-6 stimulate pathways like NF-κβ and IL-6/STAT3 signaling, which in turn promote cell survival and proliferation, with a 30 % higher chance of developing adenocarcinoma, IL-6/STAT3 signaling, and Bcl-2, supporting the inflammation-cancer transformation theory. Reactive oxygen species (ROS) and reactive nitrogen intermediates (RNI) are produced by inflammatory cells, such as neutrophils and tumor-associated macrophages (TAMs), which results in oxidative DNA damage. For instance, G-to-T transversions, a mutation common in colorectal cancer, are caused by ROS-induced 8-oxoguanine lesions. Through hypoxia-induced VEGF production *via* HIF-1α, the TME also promotes angiogenesis, creating new blood vessels to carry oxygen and nutrients. VEGF overexpression in renal cell carcinoma correlates with tumor stage and results in a 50 % increase in microvessel density (Bhol et al., 2024; Mittal et al., 2014).

To promote invasion and metastasis, cancer cells alter the extracellular matrix. Collagen and laminin are broken down by matrix metalloproteinases (MMPs), including MMP-2 and MMP-9, which permits local invasion. Overexpression of MMP-9 increases the likelihood of regional dissemination in melanoma. By upregulating vimentin and downregulating E-cadherin, the epithelial-to-mesenchymal transition (EMT), which is triggered by transcription factors such as snail and twist, improves cell motility. EMT is essential for metastasis because it enables cancer cells to enter the blood or lymph vessels, survive circulation, and spread to other locations. 40 % of metastasis cases of breast cancer include circulating tumor cells (CTCs) with EMT markers, which frequently colonize the lungs (van Zijl et al., 2011; Yayan et al., 2024).

Cancer is characterized by immune evasion, which allows tumors to elude immune monitoring. PD-L1, which binds to PD-1 on T-cells and inhibits their cytotoxic function, is upregulated by cancer cells. 50 % of melanoma patients had PD-L1 overexpression, which lowers 5-year survival by 20 %. Additionally, tumors emit immunosuppressive cytokines, including TGF-β and IL-10. Which inhibit dendritic cells and NK cells (Kim and Cho, 2022). TAMs with an M2 phenotype also release IL-10, which suppresses immune responses. Treg infiltration is associated with a 30 % poorer prognosis in pancreatic cancer, demonstrating how regulatory T-cells (Tregs) build up in the TME and inhibit effector T-cells. Oncoproteins E6 and E7 downregulate MHC class 1 in virus-associated malignancies, including HPV-driven cervical cancer, which further facilitates immunological evasion by lowering tumor visibility to T-cells in 70 % of instances (Tomaić, 2016).

Metabolic reprogramming promotes the quick development and survival of cancer. Despite oxygen availability, the Warburg effect causes cancer cells to choose glycolysis over oxidative phosphorylation, which supplies energy and metabolic precursors for growth. MYC and HIF-1α cause this change, which results in lactate production, which acidifies the TME and encourages invasion (Tufail et al., 2024). The glycolytic enzyme LDHA is increased in 90 % of triple-negative breast cancer patients, which is associated with more aggressive illness. As demonstrated by MYC-driven malignancies such as Burkitt's lymphoma, where glutamine intake doubles, cancer cells depend on glutaminolysis, upregulating glutamine absorption to feed the TCA cycle. This metabolic flexibility allows tumors to flourish in nutrient-scarce conditions, such as hypoxic tumor cores, where glycolysis supports ATP generation (Feng et al., 2018a, Feng et al., 2018b).

Therapy resistance, tumor heterogeneity, and recurrence are all influenced by cancer stem cells (CSCs). Because they can differentiate and self-renew, CSCs, which are recognized by markers like CD44 and ALDH1, can withstand therapies due to improved DNA repair and drug efflux *via* ABC transporters. In glioblastoma multiforme (GBM), CSCs comprise 1–5 % of the tumor cells, although 60 % of cases return after treatment. In leukaemia, CXCR4 suppression lowers CSC survival by 40 %. This is an example of how CSCs interact with the TME. Stromal cells secrete SDF-1, which activates CXXR4 on CSCs and promotes their survival (Kapoor-Narula and Lenka, 2022; Phi et al., 2018).

The development of cancer is coordinated by signaling pathways. 40 % of malignancies have the PI3K/AKT/mTOR pathway active, which increases protein synthesis and prevents apoptosis to promote survival. PI3K mutations raise treatment resistance in ovarian cancer, which lowers 5-year survival by 20 % (Rascio et al., 2021). By upregulating MYC and cyclin D1, the Wnt/β-catenin pathway, which is dysregulated in 80 % of colorectal tumors due to APC mutations, promotes stemness and metastasis. Pathway crosstalk increases oncogenic signaling and raises the risk of malignancy by 30 %. For example, in *H. pylori*-induced gastric cancer, IL-6/STAT3 activates PI3K/AKT (Vallée et al., 2021).

## 3 Different types of nanosponges (NSs)

Nanotechnology has emerged as an up-and-coming area of research and application, owing to its distinctive properties inherent at the nanoscale. This field encompasses many applications across various disciplines, leveraging materials' unique characteristics when manipulated at the nanoscale level (Kumar et al., 2024). One such application is the development of NSs, solid, crosslinked, mesh-like polymeric structures with a small surface area at the nanoscale. The structure has in common with all water molecules affinity, which results in supramolecular-3-dimensional-hyper-reticulated nano-sporous structures exhibiting a very high degree of stability at different pH and temperature ranges (Anwer et al., 2022a, Anwer et al., 2022b; Datta et al., 2024). The concept of “Nanosponges” was first introduced in 1994 by Wang and colleagues, who synthesized stannic oxide-based nanostructures using the sol-gel method. During their characterization using an electron microscope, they noted the formation of porous, sponge-like architectures, leading to the coining of the term (Dazhi et al., 1994). In 1996, Davankov and his team introduced the first polymer-based NSs, specifically polystyrene-based structures, which exhibited particle sizes in the 12–17 nm (Davankov et al., 1996). The earliest report on cyclodextrin (CD)-based NSs was published by Ma and Li in 1999, marking a significant advancement in the development of this class of nanocarriers (Gowda et al., 2024). In 2006, Cavalli and collaborators were the first to introduce CD-NSs as a platform for the sustained release of both hydrophilic anticancer drugs like doxorubicin (DOX) and lipophilic medicaments such as dexamethasone and flurbiprofen (Davankov et al., 1996). In 2010, Torne and team introduced paclitaxel-loaded CD-NSs to improve the oral bioavailability of the drug (Torne et al., 2010). Then came two firsts in 2013, the emergence of gold-based NSs and the development of the first biomimetic NSs reported by Hu et al. (Lin et al., 2013). The first polyester-based NSs were introduced in 2014 by Stevens and colleagues, who demonstrated effective oral and intravenous delivery of paclitaxel (Stevens et al., 2014). In 2016, Su et al. introduced the first inorganic lipo-graphene NSs platform for chemo-photothermal therapy in brain cancer (Su et al., 2016). The above timeline reflects an exciting evolution in this space, the increasing versatility and promise of NSs in various biomedically related applications.

NSs significantly improve the solubilization capacity of both water-soluble and lipid-soluble drugs. They enhance the bioavailability of drugs and enable a sustained release profile. These unique nanostructures, characterized by internal hydrophobic chambers and external hydrophilic branching, allow amphiphilic materials to encapsulate and deliver therapeutic molecules that are both hydrophilic and hydrophobic (Garg et al., 2024). The NSs' dimensions typically range from 12 to 300 nm and can exist in crystalline and paracrystalline forms. The structural form largely depends on the synthesis conditions, including the reaction environment and the materials used during processing. The crystallization abilities of NSs are of paramount importance, as they influence and govern how much drug NSs may accommodate (Davankov et al., 1996; Gore et al., 2022). Furthermore, these nanostructures are thermally stable up to 130 °C and maintain structural integrity across a wide pH range (pH 1 to 11). They are porous, biodegradable, and nontoxic, contributing to their suitability for biomedical applications (Garg et al., 2024). NSs offer several advantages, depending on the material and method of preparation. To sum up, NSs are biocompatible, non-toxic, and biodegradable. NSs are relatively simple to fabricate, have controlled and sustained release, and enhance the solubility of the drug. Additionally, they have long-term stability, scalability, and applicability for diagnosis and treatment, further underscoring their versatility as a drug delivery platform.

**Fig. 2:**
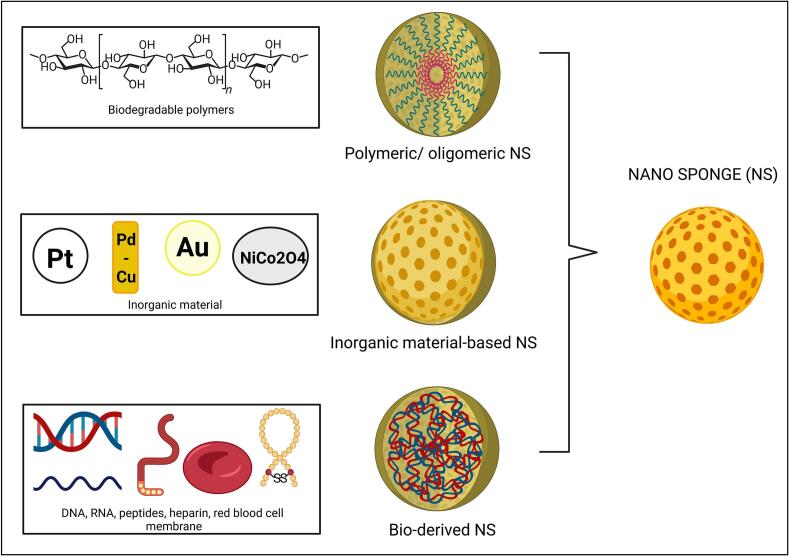
Illustration discussed the different types of nanosponges (Created in BioRender. MCOPS, P. (2025) https://BioRender.com/d05v923).

CD is a widely utilized material for the construction of NSs. These cyclic oligosaccharides are produced from glucose monomers linked by α-1,4 glycosidic linkage, resulting in a characteristic truncated cone shape. NSs of CD are considered CD cross-linked NSs. Hexamethylene diisocyanate, diphenyl carbonate, diaryl carbonate, diglycidyl ethers, and pyromellitic dianhydride are crosslinking agents fabricating CD-NSs. Citric acid has recently been examined as a greener, safer crosslinking agent (Gowda et al., 2024). The level of crosslinking influences the capacity for drug loading, as it determines the amount of void space within the NS available for this purpose. If the crosslinker disintegrates quickly, it increases the risk of releasing the dosage too rapidly (Gupta et al., 2024b; Tiwari and Bhattacharya, 2022). NSs are considered more patient-compatible due to their ability to deliver medications in a controlled and targeted manner. Furthermore, the ultra-small pore sizes provide inherent resistance to bacterial infiltration, allowing them to function as a self-sterilizing carrier, an added advantage in ensuring safe and effective drug delivery as compared to other nanocomposites (Rajam and Muthukumar, 2021). They also offer versatility in converting liquid formulations into solid forms. Also, depending on attachment or degree of polymer cross-linking, the particle size of NSs can be precisely controlled while creating variation for drug delivery applications (Kaur and Kumar, 2019). NSs can be produced more cost-effectively than several other nanocarriers, such as niosomes, ethosomes, liposomes, and solid lipid nanoparticles (Nandi and Biswas, 2024). The different types of NSs are discussed below and shown in Fig. 2.

### 3.1 Polymer/oligomer nanosponges

Polymeric NS drug delivery systems are a novel, highly structural and functional class of nanocomposites. The drug carrier systems are nanoscale carriers with a porous structure capable of entrapment of a multitude of therapeutic agents. Crosslinking biodegradable polymers together forms a stable 3D network that can encapsulate hydrophilic and lipophilic drugs at the same time to fabricate an NS. NSs, generally 1–2 nm, can cross biological barriers, such as the blood-brain barrier (BBB). The distinct ability to target drug delivery, achieve sustained release, utilize improved solubility and bioavailability, and employ a range of drugs for encapsulation potential are features not previously described for drug delivery systems. Not only do NSs have unique functional properties, but they also have high drug loading capacities and a self-sterilizing characteristic (Moin et al., 2020; Tiwari and Bhattacharya, 2022).

Abou Taleb et al. fabricated two CD-NS formulations to improve the aqueous solubility of quercetin (QCT), aiming to enhance its biological activities, specifically its antiproliferative effects and anti-SARS-CoV-2 potential. The prepared QCT-loaded CD-NS formulations demonstrated high encapsulation efficiency (EE%) of QCT (94.17–99.31 %) and particle sizes within the nanoscale range. Formation of QCT-loaded CD-NS was confirmed using FT-IR spectroscopy. At the same time, X-ray powder diffraction (XRPD) analysis revealed partial amorphization of QCT in the β-CD-based formulation (QCT-BCD/DPC 1:3) and complete amorphization in the hydroxypropyl-β-CD-based formulation (QCT-HPBCD/DPC 1:3). Scanning electron microscopy (SEM) revealed a sponge-like morphology for both formulations. The *in vitro* release studies demonstrated enhanced QCT dissolution from the developed CD-NS formulations, which exhibited significantly lower IC_50_ values against the A549 lung cancer cell line, with improvement ranging from 1.57 to 5.35-fold. Similarly, for anti-SARS-CoV-2 activity, the IC_50_ values of the QCT-loaded formulations were 595 to 26.95-fold lower than free QCT. Notably, the QCT-loaded CD-NS employing QCT-HPBCD/DPC 1:3) demonstrated superior performance in terms of *in vitro* release, antiproliferative activity, and anti-SARS-CoV-2 activity compared to the QCT-BCD/DPC 1:3. This enhanced efficacy was attributed to the superior aqueous solubility and wetting properties of hydroxypropyl-β-CD (Abou Taleb et al., 2022; Gupta et al., 2024b).

Ahmed and team successfully prepared brigatinib-loaded NSs (BGNS) and used an ultrasonic-assisted emulsion solvent evaporation technique. Among eight formulations, BGNS5 (BG: 180 mg, EC: 400 mg, PVA: 40 mg) was optimized based on its characterization, such as particle size (261 nm), PDI (0.301), zeta potential (− 19.83 mV), encapsulation efficiency (85.69 %), and drug loading (17.69 %). FTIR and DSC analyses confirmed no physicochemical interaction between the drug and polymer, while RD indicated drug amorphization within the polymer matrix. SEM images revealed spherical, porous NSs. *In vitro* drug release demonstrated sustained over 12 h, with BGNS5 releasing 62.35 % of the drug in the first 5 h, compared to 86.91 % for pure BG suspension. Release kinetics followed the Higuchi model with anomalous non-Fickian diffusion. MTT assay results showed dose-dependent cytotoxicity against A549 lung cancer cells, with BGNS exhibiting significant anticancer activity and lower cell viability (18.48–22.98 %) at higher concentrations (12.5–50 μg/mL). Haemolysis testing confirmed the biocompatibility of BGNS5, and stability studies (ICH guidelines) indicated no significant changes in particle size, %EE, or %DL over 12 weeks (Ahmed et al., 2020).

### 3.2 Inorganic-based nanosponges

Inorganic material-based NSs are synthesized using various modified methods and have been widely studied for diverse applications, including sensing, catalysis, fuel cells, actuators, electrodes, drug delivery, and cancer therapy. NSs include nickel‑cobalt oxide, titanium dioxide, gold, platinum, palladium‑copper, and magnetic materials. Compared to polymer-based NSs, inorganic NSs offer significant advantages in therapy and imaging due to their intrinsic properties. These NSs can be loaded with therapeutic agents for targeted delivery and are especially beneficial for photothermal therapy. An example of this is magnetic NSs, which allow for externally controlled delivery of a drug for precise on-demand administration, as well as serving as contrast agents for magnetic resonance imaging (MRI) (Gowda et al., 2024). In addition to the advantages listed previously, inorganic-based NSs have stability at various pH levels (1−11) and temperatures (up to 3000 °C), allowing them to remain stable in many different environments (Gupta et al., 2024c; Kudarha et al., 2024; Tiwari and Bhattacharya, 2022).

Sue et al. formulated a novel drug delivery system utilizing sponge-like carbon materials on graphene nanosheets (graphene NSs, GNS) supported by liquid bilayers (lipo-GNS). This offers a promising solution for targeted cancer therapy. This system addresses key challenges of tumor treatment, including limited penetration and insufficient cargo delivery to deep tumor regions. The lipo-GNS platform serves as both a photothermal agent and high-capacity cargo carrier, releasing therapeutic agents- docetaxel (DTX) and gasified perfluorohexane (PFH) – along with intense heat upon near-infrared (NIR) irradiation. The *in vitro* photolytic study demonstrated that Lf-lipo-US-GNS loaded with DTX and PFH, combined with NIR irradiation, effectively destroyed RG2 tumor spheroids through photothermal heating, gasification, and chemotherapy synergistic effects. The formulation showed low carrier toxicity but achieved a significant reduction in cell viability (down to 2 %) under optimized conditions. The study in nude mice demonstrated that 40 nm Lf-lipo-US-GNS showed superior tumor accumulation (200-fold higher than the larger variant) and deep penetration compared to larger NSs, aided by size effects and lactoferrin targeting. Incorporation of PFH with NIR irradiation further enhanced tumor penetration and destruction, with effective localization even in orthotopic brain tumors. Upon NIR laser treatment, the combination of DTX and PFH within the lipo-GNS effectively ruptured and suppressed xenograft tumors for around 16 days, with no recurrence observed over 60 days and no harm to surrounding tissues. This approach highlights the potential of lipo-GNS as a sophisticated platform for tumor penetration, photo-responsive therapy, and combined chemo-thermotherapy, with application in broader biomedical fields (Su et al., 2016).

Golubeva et al. worked on the optimal conditions for synthesizing hydrothermal aluminosilicates with a kaolinite structure (Al_2_Si2O_5_(OH)_4_), and NS morphology had been established. For the first time, a one-stage production method for aluminosilicate NSs with a high specific surface area (470–500 m^2^/g) has been achieved without using organic or chemical modifiers. These NSs demonstrated efficient adsorption of positively and negatively charged ions from aqueous solutions, outperforming natural kaolinite and other synthetic aluminosilicates in sorption capacity. A unique feature of the synthesized NSs is their pH-sensitive surface zeta potential, which shifts from −22 mV in alkaline conditions (pH 12) to +12 mV in acidic conditions (pH 2.5). This pH responsiveness differentiates them from CD-NSs, which typically maintain a positive zeta potential, inhibiting their applications. Hemolytic activity studies revealed that these NSs were non-toxic to human red blood cells, causing no erythrocyte damage at 0.1 to 1 mg/g concentrations. This case study highlighted the potential of aluminosilicate NSs as a versatile material for ecological and medical applications for cancer, including effective sorbents and pH-responsive drug delivery systems (Golubeva et al., 2021).

Tingting Zheng et al. prepared DOX-loaded gold nanoparticles conjugated with copolymer liposome bilayer and functionalized with RNA aptamer (DOX@GNS-PLB-Apt) to treat breast cancer. The study demonstrated that DOX@GNS-PLB nanoconstructs provided stable, long-term drug encapsulation with minimal release (∼15 % in 24 h) under neutral conditions. At the same time, acidic pH and thermal stimuli (at PLB phase transition temperatures of 45–51 °C) triggered significant DOX release. This dual pH- and thermo-responsive behaviour enabled precise, stimulus-controlled drug release for extended periods. The study showed that DOX@GNS-PLB-Apt nanoconstructs enabled pH-triggered and NIR-enhanced intracellular DOX release in MCF-7 breast cancer cells, with fluorescence confirming lysosomal localization. Compared to control and EpCAM-low/negative cells, MCF-7 cells exhibited strong drug uptake and release, leading to elevated ROS generation and apoptosis, which was further amplified by combined chemo-photothermal treatment. Cytotoxicity assays showed that DOX@GNS-PLB-Apt induced dose- and time-dependent cell death in MCF-7 cells, with significantly higher efficacy than DOX@AuNCs-PLB due to aptamer-mediated targeting and uptake. The combined chemo-photothermal treatment further enhanced apoptosis, as MTT and Annexin V-FITC/PI flow cytometry analyses confirmed. *In vivo* studies on MCF-7 tumor-bearing nude mice demonstrated that DOX@GNS-PLB-Apt nanoconstructs showed high tumor accumulation with minimal renal toxicity, while combined chemo-photothermal therapy eliminated tumors within 15 days. Histological analysis confirmed extensive tumor necrosis without significant damage to healthy organs, indicating strong therapeutic efficacy and safety. Overall, this study highlights DOX@GNS-PLB-Apt nanoconstructs as a highly effective and biocompatible platform for breast cancer therapy, offering targeted delivery, controlled drug release, and potent synergistic chemo-photothermal effects (Zheng et al., 2016).

### 3.3 Bio-derived nanosponges

Bio-derived NSs are porous structures that are fabricated by cross-linking natural biopolymers. Since NSs are created utilizing physiologically derived components such as DNA (Wang et al., 2024a, Wang et al., 2024b), RNA (Han et al., 2017), peptides (Yapa et al., 2025a, Yapa et al., 2025b), heparin (Choi et al., 2017), red blood cell membrane (Chhabria and Beeton, 2016) And other bio-derived material-based NSs are distinct from other NSs.

A peptide NSs with low polydispersity was spontaneously formed from trigonal supramolecular building blocks in aqueous buffers by Yapa et al. These structures consist of oligopeptides (5–20 residues) with either cationic or anionic properties and a hydrophobic region to form interspersed hydrophilic and hydrophobic nanodomains. Unlike traditional liposomes or vesicles, these NSs were efficiently internalized by mammalian cells. The bioavailability of perillyl alcohol (POH) (the GBM therapeutic agent that has shown limited efficacy due to poor solubility) was enhanced through the use of two types of peptides, NSs (D-POH)_10_K_20_ and (D-POH)_10_R_20_. Each structure has ten aspartates bound to POH through ester linkage, using either 20 lysine (K) or 20 arginine (R) as the positive part of the oligopeptide. Characterization by DLS and AFM revealed distinct size distributions and zeta potential for the two NS types. Cell culture studies on murine glioma cells (GL26) and neural progenitor cells (NPC) demonstrated varying efficacy in killing cancer cells and affecting NPC, with (D-POH)_10_K_20_ showing superior potential for GBM cyto-therapy. A dose-dependent decrease in cell viability was observed at 24 and 48 h. (D-POH)_10_K_20_ showed the LC50 (the concentration required to kill 50 % of cells) was very low, measured at 0.075 μg/mL at 24 h and 0.068 μg/mL at 48 h, indicating high potency against the glioma cells. The (D-POH)_10_K_20_ NSs showed significantly lower toxicity towards the non-cancerous NPC cells. A modest decrease in viability was only observed at the highest concentrations tested. The LC50 value for NPCs after 48 h was 0.614 μg/mL, nearly nine times higher than for the cancer cells. These findings establish (D-POH)_10_K_20_ as a promising candidate for cell-mediated GBM treatment (Yapa et al., 2019).

DNAzymes were recognized as promising tools for silencing disease-related genes, but their clinical use had been limited by poor stability, weak catalytic activity *in vivo*, and inefficient delivery. In this work, Dan Luo and team developed programmable DNA NSs that acted as safe and intelligent carriers, assembled in a single step to hold DNAzyme, its cofactor precursor, targeting aptamers, and a photosensitizer. These dynamic nanostructures remained stable in the body, selectively accumulated in tumors, and responded to the local microenvironment by enhancing DNAzyme activity, generating oxygen, and producing reactive oxygen species for improved photodynamic therapy. Flow cytometry analysis showed that the combined gene therapy and photodynamic therapy (ADMP + Laser) induced the highest apoptosis in 4 T1 cells, with a rate of 29.04 %. Photodynamic therapy alone (AmutDMP + Laser) caused 21.51 % apoptosis, while gene therapy alone (ADM) led to 11.11 %. In contrast, the PBS control with laser showed negligible apoptosis (0.07 %). These results highlight the superior efficacy of the combined treatment. The antitumor study over 15 days showed that the ADMP + laser group achieved the most potent inhibition, with tumor volumes remaining below 150 mm^3^ compared to nearly 600 mm^3^ in the PBS control. Moderate suppression was observed in other groups. Tumor weight analysis further confirmed these findings, with the ADMP + laser group averaging ∼0.05 g *versus* ∼0.38 g in the PBS control. The ADM and AmutDMP + laser groups showed intermediate weights of ∼0.28 g and ∼ 0.12 g, respectively (A- Aptamer, mutD- mutated DNA, M-MnO_2_, P- Photosensitizer). Together, these effects led to potent gene silencing and cancer cell killing in both cell and animal models, with minimal harm to healthy tissues. This platform demonstrated a flexible and clinically relevant strategy for delivering nucleic acid drugs and could accelerate the translation of DNAzyme-based therapies (Luo et al., 2022).

## 4 Fabrication methods of nanosponges

### 4.1 Melt method

**Fig. 3:**
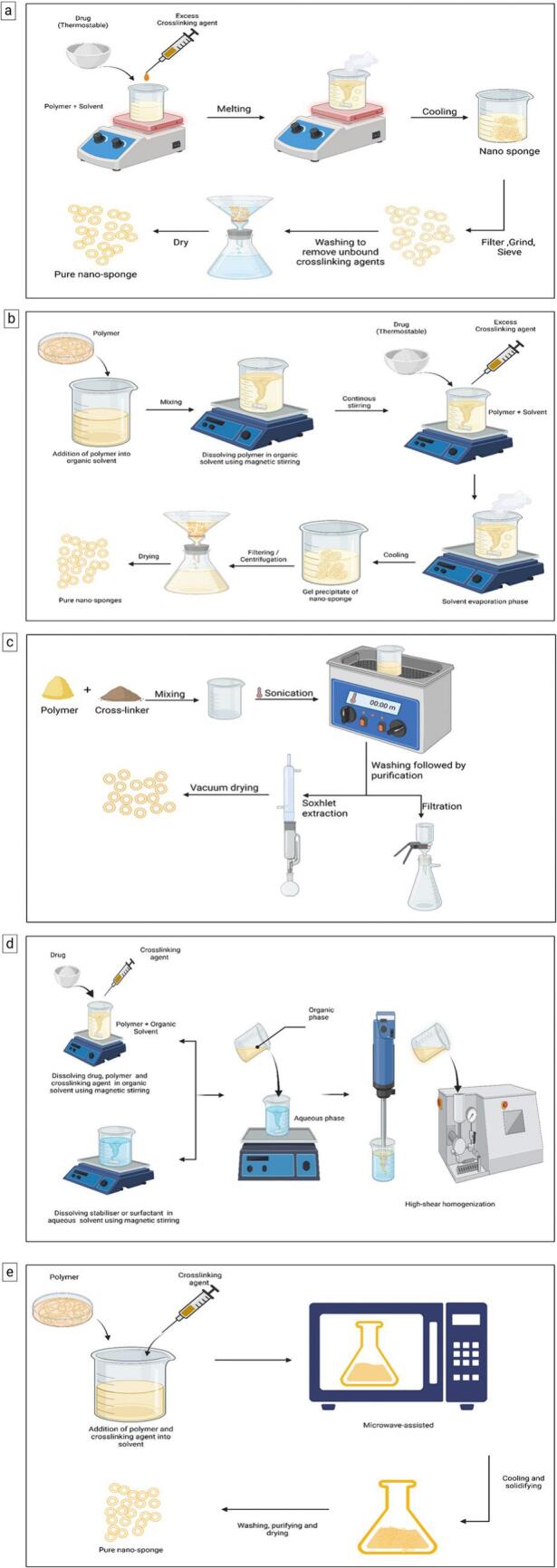
Fabrication methods of NSs a) Illustration depleted method of fabrication for NSs using melt method. b) Pictorial representation of fabrication for NSs using the solvent method. c) Pictorial representation of fabrication for NSs using Ultrasound-assisted method. d) Illustration depicts the process of fabricating NSs *via* the emulsion solvent evaporation method, highlighted the key steps involved e) Illustration showed the fabrication of NSs using a microwave-assisted method (Created in BioRender. Dhas, N. (2025) https://BioRender.com/8fdbi0x).

The melt method, which is easy and quick for the production of porous structures suitable for drug delivery systems, is an established method in the literature for the synthesis of NSs. The polymer of choice is usually CD, while the small crosslinker can vary from dimethyl carbonate to isocyanates based on compatibility and desired properties of the end product. The melting procedure amounts to heating the polymer and the crosslinker together (at about 100 °C) until they have melted and made a homogeneous mixture. Appropriate homogenization is required to obtain better crosslinker distribution in the polymer matrix for the NSs to possess uniform characteristics. When cooled, the mixture solidifies into a porous structure as crosslinked networks are formed between the chains of the polymer. The solidified material is then purified by repeatedly washing with a solvent or a solvent mixture (water and ethanol) to remove unreacted raw materials and by-products, ensuring the final product's purity. Following purification, NSs can be loaded with the drug, where the therapeutic agents are encapsulated/adsorbed/ adsorbed on or within the porous structure to enhance the drug delivery efficiency (Kadian et al., 2023; Sharma et al., 2022a, Sharma et al., 2022b). The detailed procedure for the fabrication of NSs using the melt method is shown in Fig. 3 (a).

### 4.2 Solvent method

Solvents and crosslinkers are thus utilized to establish stable NS network systems. Polymers, such as CDs, PVA, ethyl cellulose, and other degradation materials, are typically employed. Polar aprotic solvents, including DMF and DMSO, allow for the dissolution of polymers with their relative efficiency. Initial polymer dissolution in a suitable solvent established a polymer solution, which was made to react with an excess of a crosslinker-type carbonyl compound, usually dimethyl carbonate or carbonyldiimidazole. Depending on the type of NS properties sought after, the polymer to crosslinker molar ratio is kept in the range of 4:16–8:2. The mixtures were maintained at a specific temperature, up to 10 °C, and the reflux temperature of the solvent, for a period ranging from 1 to 48 h to attain crosslinking in the formation of the 3D NS network. After cooling down to room temperature and adding double-distilled water to precipitate NS, his further NS was collected through filtration and washed with water or ethanol for complete elimination of unreacted materials and residual solvents, further leading to a stable end product after drug encapsulation in the final step (Bhowmik et al., 2018, Kaur and Kumar, 2019). The detailed procedure for the fabrication of NSs using the solvent method is shown in Fig. 3 (b).

### 4.3 Ultrasound-assisted method

Ultrasound-assisted methods for NS synthesis are solvent-free, environmentally friendly techniques, where ultrasound waves assist in crosslinking of the polymer. This method is efficient, able to achieve homogenous nanostructures, and has a minimum environmental impact. The most common polymers used are β-CD, ethyl cellulose, or other types of biodegradable polymer, whereas crosslinkers are mostly diphenyl carbonate (DPC) or other organic carbonates. The common practice in this method is to mix the polymer and crosslinkers in a flask without any solvent in a given molar ratio. The flask is immersed in an ultrasonic bath at around 90 °C for about 5 h, providing the energy to drive the crosslinking reaction and form a three-dimensional network that embeds the cellulose nanocrystals. After sonication, the mixture was allowed to cool to room temperature, while the product was washed with water to remove unreacted material. Additional purification may involve soxhlet extraction using solvents like ethanol to ensure the complete removal of residual components. The purified NSs are dried under vacuum or at low temperature to yield a stable powder suitable for subsequent drug-loading applications (Gulati, 2023; Jasim et al., 2020). The detailed procedure for the fabrication of NSs using the ultrasound-assisted method is shown in Fig. 3 (c).

### 4.4 Emulsion solvent evaporation

This process creates NSs with the ideal properties for drug delivery applications by merging the concepts of emulsification with solvent evaporation. Ethyl cellulose and CDs are examples of frequently used polymers. These substances provide the NSs with their structural support. Usually, the API and polymer are dissolved using organic solvents like dichloromethane (DCM). Some organic solvents are used to dissolve the medication and polymer. This combination creates the dispersed phase, which includes the polymer and the API. Stabilizers such as Tween 80 and PVA are dissolved in the aqueous continuous phase. The emulsion that is created during mixing is stabilized during this phase. Using a magnetic stirrer, the dispersed phase is gradually introduced dropwise to the continuous phase while being forcefully stirred (often at 1000 rpm). The organic phase is distributed throughout the aqueous phase of the emulsion produced by this method. The emulsion is mixed for several hours to remove all the organic solvents. The NSs are solidified by the precipitated polymer and evaporating solvent. The NSs are filtered or centrifuged out once the organic solvent is evaporated. After obtaining the NSs, they are filtered and washed in water to remove any residual solvent or unreacted products. To ensure that all moisture and solvent are removed from the NSs, the NSs are vacuum dried or dried at low temperature (40 °C) to produce stable nanostructures (Ahmed et al., 2021; Dhanaraju et al., 2024). The detailed procedure for the fabrication of NSs using the emulsion solvent evaporation method is shown in Fig. 3 (d).

### 4.5 Microwave-assisted method

The microwave-assisted method is an inventive and effective strategy for fabricating NSs, which uses microwave irradiation to simulate polymer crosslinking. This method has advantages over conventional methods, including faster reaction rates, higher yield, and better homogeneity of resulting NSs. When synthesizing an NS, β-CD, a frequently used polymer, is a key component of NSs because it can form inclusion complexes with various drugs. DPC is often used as a crosslinker to generate an NS structure. The process is scientific, simply requiring the polymer (β-CD) and crosslinker (DPC) to be delivered in their proper molar ratios, and in a solvent (dimethylformamide, DMF) in a reactor flask. The polymer and crosslinker mixture receives 2450 MHZ of microwave irradiance. Microwave irradiation directs homogeneous heating across the entire reactor mixture, determining whether the polymer chains can be crosslinked rapidly. By optimizing temperature and reaction time, one can achieve desired characteristics of the final product using processes, such as Box–Behnken design, for the polymer formulation. Solid NSs are fabricated by removing solvent *via* distillation after the predetermined reaction time has lapsed. As a preliminary clean-up of unreacted compounds or leftover solvents, the resulting NSs are cleaned with water and refined further using Soxhlet extraction with ethanol. To verify the effective synthesis and assess the physical characteristics of the NSs, the finished product is evaluated using methods including Scanning Electron Microscopy (SEM), Fourier Transform Infrared Spectroscopy (FTIR), and Differential Scanning Calorimeter (DSC) (Sharma et al., 2022a, Sharma et al., 2022b; Tiwari and Bhattacharya, 2022). The detailed procedure for the fabrication of NSs using the microwave-assisted method is shown in Fig. 3 (e).

### 4.6 Photolytic templated assisted method

The photolytic templated method is an advanced strategy for producing porous structures with a high surface area. In this case, photolytic materials were used to prepare NSs. The technique utilizes sacrificial templates removed after the NSs have been formed and allows for fabricating materials particularly suited for applications such as medication delivery or environmental remediation. The first step in the method is the development of the sacrificial template, which determines the structure and porosity of the final product, and it is typically made using natural-based biopolymers (cellulose) or organic materials (sugar). Then, using sol-gel processing or chemical vapour deposition, a photolytic material (TiO_2_), silver (Ag), or other metal oxide composites is created and applied to the template. The photocatalyst is activated by further light irradiation, which triggers photolytic processes that encourage the creation of a solid matrix surrounding the template or the crosslinking of polymer chains. The template is eliminated by dissolving it in water or another appropriate solvent after a sufficient photocatalytic reaction and structural solidification, leaving behind a porous NSs structure. This method provides a flexible framework for creating high-performance functional material NSs (Taha et al., 2024, p. 3; Yu et al., 2012).

## 5 Mechanism of action of the nanosponge as a carrier

Nanosponge represents a novel cancer therapy platform that improves drug delivery, efficacy, and safety through structural and multifunctional properties. The sponge-like structure with interconnecting pores is suitable for encapsulation of a diverse therapeutic agents: proteins, nucleic acids, and chemotherapeutics, with biodegradable and biocompatible small-molecule materials such as CDs, PVA, or other polymers (Jha et al., 2024a, Jha et al., 2024b). Even though a controlled release mechanism enables prolonged drug delivery within therapeutic levels to the tumor and avoids systemic exposure, the porous structure enhances adsorption and loading of hydrophobic drugs, addressing solubility issues. NS carriers can be designed for a more targeted mechanism of delivery by functionalizing the surface with ligands, such as peptides or antibodies, and binding to receptors that are overexpressed on the cancer cell, utilizing the enhanced permeation and retention (EPR) effect of the tumor vasculature, while limiting damage to healthy tissue and improving drug delivery. The stability for therapeutic implementation is enhanced because the time and bioavailability of the drug are prolonged by making the active agents almost inert to rapid elimination in circulation. Drug-loaded nanocarriers-electrospun NS DOX (DOX), for example-are believed to interact with cancer cells at the tumor site through endocytosis to allow for further direct cytoplasmic delivery of drugs and resultant cell death. Combination therapies provide examples of their multifunctional applications, wherein they co-deliver chemotherapeutics with gene therapies or integrate modalities such as photothermal treatment to enhance therapeutic efficacy. Additionally, certain NSs have biosensing features to track therapeutic effects in real time. Recent developments demonstrate their potential, including formulations that improve drug solubility and stability while exhibiting vigorous anticancer activity in preclinical studies, ethylcellulose NSs for sustained release of abemaciclib (Teja and Radha, 2023), and DNA-based NSs for co-delivering DOX and antisense oligonucleotides to regulate genes (Mohite et al., 2024). These characteristics of NSs are a game-changing innovation in cancer treatment, offering improved results and fewer adverse effects (Gowda et al., 2024; Kumar and Bhowmik, 2019).

## 6 Nanosponges as emerging carriers in Cancer therapy: unlocking Tumor targeting and uptake

Novel drug delivery technologies that maximize therapeutic efficacy and reduce adverse effects have been introduced by nanotechnology, transforming many industries, including pharmaceutical companies (Chen et al., 2024). Between these developments, NSs have shown promise as a vehicle for anticancer drugs (Koppula and Maddi, 2024). In-depth discussion of the benefits of NSs in this situation will be provided, along with an emphasis on the special qualities and mechanisms that enhance the effectiveness of cancer treatments (Garg et al., 2023; Gupta et al., 2023).

### 6.1 Targeted drug delivery to the cancer cells

The potential of NSs to provide targeted medicine delivery is one of their most significant benefits. Because the drugs used in traditional chemotherapy are distributed non-specifically throughout the body, they frequently cause systemic toxicity (Garg et al., 2023; Gupta et al., 2025b; Tiwari and Bhattacharya, 2022). This focused method minimizes side effects and improves patient compliance by reducing drug exposure to healthy tissues while enhancing medication accumulation at the tumor location (Al-Thani et al., 2024). NSs particles have a particular size, and their polarity can be altered by altering the amount of crosslinking agents and polymers. NSs with variable void polarity exhibit diameters of 1 μm or less. They may be crystalline or para-crystalline. Since the degree of crystallization significantly impacts the productivity of stacking NSs, the crystalline structure of NSs is crucial for drug complexation. Paracrystalline structure has been demonstrated that NSs can load many different kinds of drugs. They stay stable at temperatures up to 130 °C and pH values between 1 and 11. They are discovered to be non-toxic, biodegradable, and porous. Because of their three-dimensional structure, they can encapsulate, transport, and provide observable release of drugs and other compounds (Garg et al., 2023). Choi et al. discussed the hybrid nanoparticle hydrogel for targeted drug delivery in biomedical applications. The author explains the purpose of hybrid NP-hydrogel systems in drug administration, how to make them, and how combining them solves specific issues (Choi and Kohane, 2024). Caldera et al. prepared the DOX-loaded polysaccharide-based NSs for the treatment of cancer. The cyclic nigerosyl-1-6-nigerose (CNN)-NSs contained DOX in a good amount. Solid-state NMR and DSC investigations demonstrated that the medication interacted with the polymer matrix. Studies conducted *in vitro* showed that the drug's delayed and extended-release kinetics relied on pH. CNN-NSs loaded with DOX were readily absorbed in the A2780 cell line. It might be regarded as an intracellular reservoir for DOX, which gradually releases the drug. CNN NSs loaded with DOX have the potential for localized cancer treatment (Caldera et al., 2018). Kapoor et al. discussed the benefits of CD-NSs for cancer management. CD-based NSs can encapsulate a high amount of active pharmaceutical ingredients and enhance the solubility of the non-polar compounds. NSs are a promising strategy for treating cancer with fewer side effects (Kapoor et al., 2024). NSs provide advantages for targeted drug delivery compared to liposomes, dendrimers, and polymeric nanoparticles because of their cross-linked porous structures that are capable of significant drug entrapment and encapsulation with controlled release at the TME. They provide easy access to achieve surface modification for active targeting, they are more stable with less premature release, and they have higher drug entrapment capacity when compared to liposomes. They have the highest drug loading capacity of all four delivery systems since dendrimers are branched structures, whereas NSs are cross-linked and have much higher entrapment capacity while providing a sustained release profile compared to dendrimers. In contrast to polymeric nanoparticles, NSs have a tunable surface and porous framework designed for increased tumor-specific accumulation by the EPR effect. As an example, CDs made NSs encapsulated with DOX show superior tumor targeting efficiencies, and lower systemic toxicities compared to conventional liposomes (Eltaib, 2025; Islam et al., 2025; Mody et al., 2014).

### 6.2 Extended drug release in the tumor environment

Nanocarriers are gaining importance for the controlled release to the cancer cells. Nanocarriers, including micelles, dendrimers, liposomes, polymeric/lipid nanoparticles, nanocrystals, and quantum dots, have become efficient methods for site-specific delivery and controlled release (Chen et al., 2020; Colaco et al., 2024). Each nanocarrier system offers distinct features and benefits by modulating the physicochemical properties, *in vitro* characteristics, and *in vivo* performance of integrated medicines (Lee et al., 2020). To maximize therapeutic advantages and minimize adverse effects, NSs have gained a lot of interest since they have been delivered directly to the tumor site. Conventional methods often attain high plasma concentrations and deliver their payload instantly, but the elimination process causes these concentrations to decrease (Zeb et al., 2022). Drug levels in the plasma fluctuate as a result, and frequent, repeated administration is required, putting the patient at greater risk for toxicity and less than ideal therapeutic outcomes. For the controlled release and targeted administration of medicines, the key objectives continue to be the short half-life, nonspecific dispersion, and off-target toxicity of traditional methods (Kamaly et al., 2016). NSs are especially useful in treating chronic conditions (cancer, arthritis, and ulcers) that must be administered repeatedly over weeks, months, or even years. Extended, protracted, sustained, timed, and modified releases are just a few of the names given to NSs. Gowda et al. discussed the use of NSs for the treatment and diagnosis of cancer. The author examined the types, general characteristics, and preparation techniques of NSs. Along with thorough explanations of the respective patents, the scope of NSs' application in treating various kinds of cancer is also thoroughly examined. Additionally, the release pattern of the NSs and the importance of NSs in phototherapy and cancer theranostics were discussed (Gowda et al., 2024). NSs can interact with numerous functional groups, demonstrating targeted release of different compounds. This ability can be modified by using synthetic linkers that target the sites. Adding magnetic properties to a configuration of NSs during manufacturing by expanding ferrite and other magnetic materials enables targeted discharge through an external magnetic field (Bolmal et al., 2013). NSs are the preferred material because of their appealing characteristics, which include the ability to encapsulate immiscible liquids, provide sustained drug release for up to 24 h, and offer greater flexibility, stability, and reduced irritation (Tiwari and Bhattacharya, 2022). Reddy et al. prepared the entrectinib (ENT) loaded NSs using the hydroxypropyl-β-CD (HP-β-CD) to treat cancer. After optimization, a molar ratio of 0.800 mg was used to construct ENT-loaded HPβCD NSPs. They were then agitated for 420 min at 3000 rpm, yielding a desirability of 0.926. Particle size (PS), polydispersity index (PDI), and entrapment efficiency (EE%) were predicted to be 146.98 nm, 0.263, and 88.29 %, respectively. The improved formulation had an EE of 87.36 ± 1.61 %, a PDI of 0.233 ± 0.049, and a mean size of 151.8 ± 5.6 nm. The effectiveness of the optimization was further validated by several studies, showing significant gains in AUC0-t (6.30-fold) and Cmax (4.10 times) as compared to the free medication (Reddy and Bhikshapathi, 2024). NSs enable efficient and prolonged drug release in tumor tissues through their porous, cross-linked framework, ensuring controlled drug delivery without the risk of burst release or reliance on polymer degradation. Unlike liposomes, prone to leakage, and dendrimers that show rapid drug release. Ribociclib loaded into NSs showed a more sustained release of over 80 %, which translated into improved tumor cell killing compared to other nanocarriers (Cheng et al., 2025; Iravani and Varma, 2022; Ray et al., 2018).

### 6.3 Enhancement of systemic bioavailability

NSs are versatile, nanoscale structures capable of solubilizing both polar and nonpolar drugs, thereby enhancing their bioavailability and enabling sustained release. Their amphiphilic nature, characterized by external hydrophilic branches and internal hydrophobic cavities, allows simultaneous encapsulation of hydrophilic and hydrophobic compounds (Garg et al., 2023; Gupta et al., 2025a). They have advantages over other nanoparticles due to their facile reproducibility through various treatments, including photothermal, photodynamic, pH-responsive or ionic strength (J. et al., 2022). Dora et al. developed the erlotinib (ERL)-loaded β-CD-NSs to enhance solubility and bioavailability. The particle size of the NSs was 372 ± 31 nm, PDI 0.21 ± 0.07, and zeta potential −32.07 ± 4.58 mV. Cell uptake studies revealed superior internalization of ERL-NS and Coumarin-6-NS in MIA-PaCa-2 and PANC-1 cells, with uptake 5.4–5.6-fold higher than free ERL. Cytotoxicity assays demonstrated significantly lower IC50 values for ERL-NS across 24–72 h compared to ERL, along with a higher apoptotic index (0.79–0.82 *vs.* 0.37–0.42). Blank nanosponges were non-toxic. *In vivo* pharmacokinetic analysis confirmed enhanced oral bioavailability of ERL-NS, showing nearly 2-fold increases in Cmax (78.98 *vs.* 42.36 μg/mL) and AUC_0−∞_ (1079.95 ± 41.38 *vs.* 580.43 ± 71.91), with relative bioavailability ∼200 %. The improved performance was attributed to inclusion complexation, reduced crystallinity, enhanced solubility, and avoidance of presystemic metabolism (Dora et al., 2016). Gigliotti et al. developed the camptothecin-loaded NSs for the treatment of cancer. The findings demonstrated that, compared to free camptothecin, β-CD-NSs camptothecin dramatically reduced the viability, clonogenic potential, and cell-cycle progression of ATC cell lines, with a quicker and more pronounced impact. Additionally, β-CD-NSs-camptothecin suppressed the production of β-PIX, a member of the Rho family of activators, migration, phosphorylation of the Erk1/2 MAPK, adherence of tumor cells to vascular endothelial cells, and the secretion of pro-angiogenic molecules (VEGF-α and IL-8) (Gigliotti et al., 2017). Pei et al. fabricated the fluorescent hyper-cross-linked β-CD‑carbon quantum dot hybrid NSs for cancer treatment. The dimensions of the NSs measured 200 nm, exhibiting commendable biocompatibility and a striking bright blue fluorescence when excited at 365 nm, accompanied by a notable photoluminescence quantum yield of 38 %. The DOX-loaded NSs showed a 300 nm size, and drug loading was 39.5 %. The NSs showed the pH-dependent drug release in a controlled manner. The NSs showed better antitumor activity than the free DOX in the HepG2 cells (Pei et al., 2018). NSs markedly enhance systemic bioavailability compared to other nanoparticles owing to their porous, cross-linked architecture that improves drug solubility, stability, loading capacity, and sustained release. Unlike liposomes with limited loading and stability issues or dendrimers with potential immunogenicity, NSs protect drugs from degradation and facilitate efficient absorption. In contrast to polymeric nanoparticles with relatively lower encapsulation efficiency, NSs form stable drug–polymer complexes that enhance bioavailability. For instance, CD-based NSs loaded with griseofulvin achieved nearly three times higher oral bioavailability than the free drug (Lu et al., 2021; Omar et al., 2020; Tiwari and Bhattacharya, 2022).

### 6.4 Enhanced tumor tissue uptake

Over the past 50 years, nanoparticle-based cancer treatment has dramatically improved the administration of chemotherapeutic medications and a variety of novel therapeutic agents, including molecular targeting agents, peptides, proteins, and genes. Nonetheless, many biological obstacles continue to impede the delivery of nanomedicines, and significant research is being conducted to surmount these obstacles (Ding et al., 2019). TMEs are the primary cause of the poor penetration of nanomedicines at the tumor sites. NSs are a drug delivery system that enhances drug penetration into tumor tissues. These nanocarriers are three-dimensional porous structures, offering unique advantages in overcoming the barriers associated with conventional drug delivery methods, especially in cancer therapy. After NSs are administered, they will flow in the bloodstream and accumulate at the target site due to the EPR effect (Iravani and Varma, 2022; Tiwari and Bhattacharya, 2022). The NSs accumulate in the tumor tissue compared to the healthy tissues because of leaky vesicles and poor drainage (Shinde et al., 2022). The NSs can encapsulate a high amount of drug and protect it from enzymatic degradation and premature clearance, which helps increase the amount of drug available in the tumor tissue. NSs help in sustained and controlled release of the drug in TME, allowing the drug to be exposed to the tumor tissue for a longer period (Afzal et al., 2022). The surface modification of NSs with peptides, antibodies, and other targeting agents can help actively target the tumor tissue. The stimuli-based drug release from the NSs in the pH and TEM conditions can also release the drug within the tumor tissue (Zhao et al., 2020). NSs also bypass the efflux pumps like P-gp, which is the main reason for drug resistance in cancer treatment. Due to these properties, NSs can help to improve the drug uptake in the tumor tissue (Xue and Liang, 2012). Trotta et al. discussed the application of NSs for cancer drug delivery. In the article, the author discusses the uses of CD-NSs as anticancer medication delivery systems. The author discussed recent clever NSs that can react to external stimuli. Results from *in vitro* and *in vivo* experiments using presently prescribed drugs, including DOX, 5-fluorouracil (5-FU), tamoxifen, and paclitaxel (PTX) (Trotta et al., 2014). NSs demonstrate superior uptake in tumor tissues compared to liposomes, dendrimers, and polymeric nanoparticles due to their optimal size range (200–300 nm), porous framework, and tunable surface properties that facilitate penetration through the EPR effect. In contrast to liposomes, which face stability limitations, or dendrimers that are rapidly cleared, NSs provide prolonged circulation and can sequester tumor-derived toxins, enhancing their tumor accumulation. For instance, ribociclib-loaded NSs demonstrated enhanced tumor uptake and greater cytotoxic activity in breast cancer models than the free drug or other nanocarriers, highlighting their improved targeted delivery and therapeutic performance (Iravani and Varma, 2022; Shah et al., 2023; Yapa et al., 2025a, Yapa et al., 2025b). The Table 1 summarizes the tumor uptake and bioavailability of anticancer drugs formulated in NSs.

**Table 1:** Summary of studies demonstrating the improved bioavailability and cellular uptake of anticancer drugs formulated in nanosponges.

Sr. No.	Composition of NSs	Tumor uptake studies	Bioavailability	Reference
1	Lapatinib ditosylate (LD)-loaded ternary CD NSs (CDNS)	F2 NS markedly enhanced cytotoxicity in MCF-7 cells with lower IC_50_ values *vs* pure LD	Oral bioavailability of LD was increased 3.34-fold with F2, with higher AUC (3.34-fold), Cmax (2.76-fold), and absorption rate constant (0.133 to 0.168/h).	(Tanaji Mane et al., 2023)
2	DOX-loaded or 6-coumarin-labeled-Glutathione-functionalized NSs;	Red fluorescence intensity ∼2× higher for DOX-GSH-NS *vs* DOX	DOX accumulation is similar for free DOX and DOX-GSH-NS at all time points; rapid uptake at 0.5 h, stable until 6 h, and decreased at 24 h	(Daga et al., 2020)
3	Lapatinib (LPT)-loaded NSs (NSs)	–	LPT-loaded NSs demonstrated significantly higher Cmax (∼3-fold) and AUC (∼2.2-fold) compared to pure LPT, with faster absorption (Tmax 3.16 h *vs* 4.33 h), confirming improved oral bioavailability	(Prabhu et al., 2021)
4	Ibrutinib (IBR)-loaded, CDI-crosslinked hydroxypropyl-β-CD (HPβCD) NSs	–	IBR-NSPs demonstrated 6.45-fold higher Cmax and 14.96-fold higher AUC_0–t_ compared to pure IBR suspension	(Sampathi et al., 2025)
5	functionalized copper-starving NSs CCM@PIT with polydopamine (PDA) and imidazole modified organosilica (IMS) nanoparticles	3.5-fold higher uptake with CCM@PIT *vs* PIT	–	(Chen et al., 2025)
6	DOX-loaded β-CD-based NSs (BNS-DOX)	Confocal microscopy showed higher intracellular fluorescence of DOX in BNS-DOX-treated cells compared to free DOX	–	(Argenziano et al., 2020b)

The overall summary of the application and advantages of NSs in drug delivery is shown in Table 2.

**Table 2:** Application and advantages of NSs in drug delivery.

Application	Advantages
Targeted drug delivery	• Reduce systemic toxicity and improve therapeutic efficacy (Al-Thani et al., 2024). • Enhance drug solubility, prolong drug release, and minimize side effects (Kapoor et al., 2024).
Enhanced solubility and bioavailability	• Improves the solubilization of hydrophilic and hydrophobic or polar and non-polar drugs. • **Easily reproducible** using **light heating, washing with eco-friendly solvents, and pH modification**. • **Higher absorption and bioavailability (Erlotinib-loaded NS)** (Dora et al., 2016). • Reducing tumor cell viability/ anticancer effect (Camptothecin-loaded NSs) (Gigliotti et al., 2017). • pH-dependent controlled release (DOX-loaded NSs) (Pei et al., 2018).
Controlled release of the drug	• **Steady and prolonged release** • **Entrectinib-loaded NSs (better bioavailability and improved efficiency)** (Reddy and Bhikshapathi, 2024).
Enhanced penetration into tumor tissue	• Improving **drug penetration into tumor tissues**. • **Enhanced permeability and retention (EPR) effect** (Iravani and Varma, 2022; Tiwari and Bhattacharya, 2022).

## 7 Multifunctionality in drug delivery

**Fig. 4:**
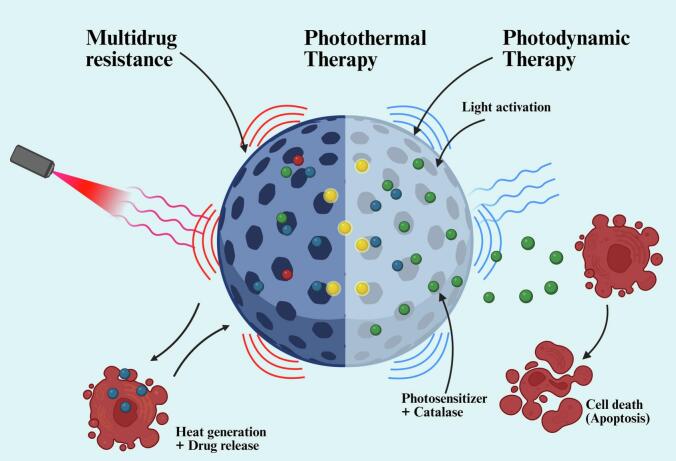
Multifunctional Nanosponge Applications in Advanced Cancer Therapy. The figure illustrates key therapeutic strategies using nanosponges, including overcoming multidrug resistance, photothermal therapy, and photodynamic therapy (Created in BioRender. Dhas, N. (2025) https://BioRender.com/gzmqdkm).

Nanosponges are a remarkably versatile platform for overcoming significant hurdles in modern medicine, particularly in the treatment of cancer and infectious diseases. They enhance treatment of multidrug-resistant (MDR) cells by encapsulating drugs, protecting them from enzymatic degradation, and bypassing efflux pumps, thereby achieving higher intracellular drug concentrations (Iravani and Varma, 2022; Lee et al., 2022). In addition to their conventional application in drug delivery, NSs facilitate advanced therapies such as photothermal and photodynamic therapies. The advanced concept of photothermal therapy (PTT) depends on NSs encapsulated with small molecules like gold nanorods (AuNRs) to convert near-infrared light into heat, thereby selectively destroying cancer cells at localized temperatures (Deinavizadeh et al., 2023; Iravani and Varma, 2022). In a similar manner photodynamic therapy (PDT) uses NSs to deliver a photosensitizer directly into the tumor and enhances drug stability and targeting in addition to providing oxygen for hypoxia in the tumor core, in turn improving therapeutic effectiveness and limiting toxicity to healthy tissue (Itoo et al., 2022; Lamy et al., 2023). The multifunctionality of nanosponges in drug delivery is depicted in Fig. 4.

NSs have emerged as a versatile and innovative platform for drug delivery, offering significant advantages in the formulation of therapeutic agents. These nanostructured carriers are characterized by their highly porous particle that encapsulate a high amount of drug, including hydrophilic and lipophilic compounds (Tiwari and Bhattacharya, 2022). NSs are appropriate for delivering several medication classes because they can hold many active pharmaceutical ingredients. Classes II and IV of the biopharmaceutical classification system (BCS) encompass poorly soluble drugs that often encounter issues with bioavailability (Garg et al., 2023). NSs can load anti-cancer drugs such as PTX and DOX, thereby enhancing their solubility and stability, which increases the penetration of the drugs into cancer tissue (Shringirishi et al., 2014). The excipient ratio, formulation parameters, and synthesis method used in the preparation of NSs can influence the release kinetics of the formulation. It also affects the porosity and degree of degradation of the NSs (Sharma et al., 2016). The sustained release of the drug from the NSs can be achieved by adjusting the crosslinking density of the NSs, which facilitates drug release at the desired pH and temperature (Paolini et al., 2019). The NSs can be functionalized using targeting ligands and antibodies to increase the selectivity for cancerous cells and tissues. This strategy will increase the drug concentration at the target site (Yoo et al., 2019). Folic acid is an example of target delivery. In this work, NSs were modified using folic acid and targeted the folate receptor, which was overexpressed on the surface of cancer cells (Ebrahimnejad et al., 2022). NSs have the advantage that they can be administered orally, parenterally, topically, and by inhalation delivery (Gupta et al., 2022). Additionally, hydrogels are suitable for topical use in wound healing (Kim et al., 2020). NSs are composed of biocompatible and biodegradable materials (Jain et al., 2020), which enhances the safety of the formulation. Natural polymers, such as CDs or chitosan, are also utilized (Mukhtar et al., 2021).

### 7.1 Minimization of anti-cancer drug resistance

The NSs are game-changers in the field of cancer treatment, particularly in cases of drug resistance (Garg et al., 2023; Iravani and Varma, 2022; Gowda et al., 2024). The drug resistance in cancer treatment is a burning issue that is caused by changes in cellular signaling pathways, overexpression of efflux pumps, and modifications in therapeutic targets. These mechanisms can significantly decrease the efficacy of chemotherapeutic drugs, potentially leading to treatment failure and unfavorable patient outcomes (Tian et al., 2024). NSs possess special qualities, including easy drug administration, improved cancer treatment, and reduced drug resistance (Tiwari and Bhattacharya, 2022). The development of drug resistance in cancer cells may occur through various mechanisms, such as the overexpression of the protein P-glycoprotein (P-gp), which effluxes chemotherapeutic drugs out of the cells (Pilotto Heming et al., 2022). Other mechanisms, such as genetic mutations that affect drug targeting or metabolic processes, alter apoptotic signaling pathways, thereby avoiding programmed cell death. Furthermore, by fostering circumstances that encourage cell survival in the face of anticancer drugs, the TME might exacerbate resistance (Liu et al., 2024). Vankudre et al. prepared the dacarbazine-loaded NSs for the treatment of melanoma. High entrapment efficiency (92.2 %) and controlled particle size (338.6 nm) were demonstrated by the optimized drug-loaded NSs. Subsequent *in-vitro* characterization of DZ NSs hydrogel revealed sustained drug release (82 % over 12 h), *ex vivo* skin penetration (73 % through sheep skin), and good *in-vivo* biocompatibility with few skin reaction indicators. With an IC_50_ value of 68.81 μg/mL, the optimized formulation demonstrated significant anti-proliferative activity in comparison to the standard cisplatin, which had an IC_50_ value of 6022.0 μg/mL, suggesting superior inhibition of melanoma cell proliferation (Vankudre et al., 2025). Mashaqbeh et al. prepared 5-fluoracil and curcumin-loaded NSs for the colon-targeted delivery. The optimized microbeads were spherical and had a mean particle size of 1.1 ± 0.05 mm. To ensure drug delivery to the colon, the generated beads were coated with a multilayer coating of ethyl cellulose and Eudragit S100 polymers, which functioned as pH-dependent and time-dependent coatings. The drug's controlled release and prolonged cytotoxic effect were demonstrated by the developed formulation (Coated MB5). Zero-order and first-order release functions best characterized the release profiles of 5-fluorouracil and curcumin from Coated MB5. For 24 h, roughly 66.4 % of 5-FU and 73.1 % of curcumin were released. Seven to nine hours after administration, the optimized coated microbeads were successfully delivered to the colon, as determined by an *in vivo* radiographic evaluation conducted in a rabbit model (Mashaqbeh et al., 2024).

### 7.2 Photothermal therapy

In cancer treatment, photothermal therapy (PTT), a minimally invasive photo-based treatment, has garnered significant interest. Using photothermal agents (PTA) and light radiation, PTT produces heat that denaturates proteins, disrupts cell membranes, and damages DNA, all destroying tumor tissue. NIR light is most frequently employed in PTT because it may penetrate deeper tissues with less scattering than visible and ultraviolet light. Polymer-based NSs have recently attracted much attention for their ability to accurately distribute PTA to the tumor site *via* active or passive targeting, resulting in improved PTT and less damage to surrounding healthy tissues (Gowda et al., 2024). Furthermore, NSs based on inorganic materials can function as PTA for efficient PTT. Even while PTT is highly recommended for eliminating tumors, it cannot eradicate the whole tumor, leaving only a small number of cancer cells, which increases the likelihood that the tumor will return. Chemo-photothermal therapy has been developed to address this problem and treat tumors synergistically and efficiently. To treat breast cancer, for example, Zheng and associates created DOX-loaded dual-stimuli-responsive gold nanoparticles (AuNPs) for targeted chemo-photothermal treatment (Zheng et al., 2016). Here, Au NSs were initially created by the authors using alloying and dealloying methods. With many holes to accommodate the medication, the created AuNPs had a diameter of around 114 ± 6.8 nm. A copolymer-liposomal layer (gatekeepers) was also applied to the drug-loaded Au nanosystems to provide them with pH and temperature responsiveness. Lastly, the nanosystem's surface is coupled with an RNA aptamer to increase cell internalization and target tumors. In acidic pH, the fabricated Au NSs showed the highest DOX release. Moreover, temperature-responsive drug delivery was made possible by the poly(N-isopropylacrylamide) pNIPAM segment in the coated layer, which changes to release DOX at 45 °C. Furthermore, during PTT, the phospholipid's hydrophobic tails changed to a perpendicular condition, mimicking the gate-opening mechanism to optimize the release of DOX from Au NSs. In comparison to DOX@Au NSs without NIR irradiation and Au NSs with NIR irradiation, the DOX@Au NSs with NIR irradiation demonstrated the most significant decrease in tumor size in breast cancer-bearing nude mice, with maximal accumulation and retention in targeted tumor cells. These findings unequivocally show that chemo-photothermal therapy based on stimuli-responsive AuNPs is an appropriate approach for successful breast cancer treatment. The synthesis and characterization of β-CD-NSs inclusion compounds (IC) containing the anti-tumor medications Cytoxan (CYT) and Melphalan (MPH) were described by Sebastián Salazar and team. Additionally, AuNPs are added to both systems to release the drugs through laser irradiation. Scanning electron microscopy (SEM), X-ray powder diffraction (XRPD), energy dispersive spectroscopy (EDS), thermogravimetric analysis (TGA), UV–Vis, and proton nuclear magnetic resonance (^1^H NMR) were used to analyze the NS-MPH and NS-CYT inclusion compounds. Thus, it was verified that MPH and CYT were present inside the NSs' cavities. A two-phase system (aqueous-organic) was used to study drug migration under laser irradiation, incorporating inclusion complexes (ICs) with AuNPs. Localized heating facilitated the release of MPH and CYT, with UV–Vis spectroscopy employed to quantify drug concentrations using the Lambert-Beer equation. Molar attenuation coefficients were 9.31 mM^−1^ cm^−1^ (MPH) and 7.27 mM^−1^ cm^−1^ (CYT). Drug release was analyzed using NSs synthesized at β-CD: DPC molar ratios of 1:4 and 1:8. Drug migration increased over time, peaking at 60 min (CYT) and 75 min (MPH). NSs (1:4) exhibited higher release efficiencies, indicating that crosslinking influences drug entrapment and release. Comparing IC-AuNPs with non-irradiated ICs and ICs without AuNPs confirmed that AuNP-induced hyperthermia significantly enhances drug migration, with no burst release, suggesting controlled drug delivery potential (Salazar et al., 2021).

### 7.3 Photodynamic therapy

Another photo-based cancer treatment is called photodynamic therapy (PDT). In contrast to PTT, PDT uses photosensitizers (PS) and incoming radiation to produce reactive oxygen species (ROS), such as singlet oxygen ^1^O_2_, which further kills the cancer cells (Dolmans et al., 2003). Nevertheless, the PS has many drawbacks, including poor stability, hydrophobicity, unpredictable photoactivity, negligible biodistribution, lack of tumor targeting, and sluggish body clearance, all of which contribute to the poor effectiveness of PDT and many adverse effects. Several NP-based carriers have gained attention to address this problem. Among them, NSs are one such carrier that has had encouraging outcomes in cancer PDT. Pan and colleagues recently described Catalase and porphyrin-loaded aptamer-conjugated DNA-based NSs for targeted PDT (Pan et al., 2020). The authors used a rolling circle amplification approach to create DNA NSs by loading porphyrin and catalase simultaneously and encoding template DNA with multiple information. By reducing hypoxic circumstances and catalyzing endogenous H_2_O_2_, the integrated catalase enzyme can produce O_2_ at the tumor site and lessen tumor resistance. Additionally, the NSs' hypoxia-inducible factor 1α (HIF1α) antisense DNA downregulates HIF1α to increase PDT sensitivity. However, when exposed to light, porphyrin produces singlet oxygen, and the aptamer aids in locating cancer cells that have an excess of tyrosine protein kinase-7 receptors. As an effective PS carrier for targeted PDT with decreased tumor resistance, the created DNA NSs, which had particle sizes between 300 and 350 nm, dramatically suppressed tumor development in mice with cervical cancer through the downregulation of HIF1α.

## 8 Biomedical applications of NSs for different types of cancer

NSs have emerged as a promising drug delivery system for cancer treatment, addressing limitations of anticancer agents, such as poor solubility, high toxicity, low stability, and limited bioavailability. Their unique porous structures allow efficient drug encapsulation, protection from degradation, and sustained release, enhancing therapeutic efficacy while reducing side effects (Gowda et al., 2024; Koppula and Maddi, 2024). NSs can be functionalized with targeting moieties to ensure drug delivery to the tumor site, improving bioavailability and efficacy even at low doses (Gowda et al., 2024). Additionally, NS fabrication and drug-loading processes are scalable, making them suitable for industrial applications (Tiwari and Bhattacharya, 2022). With advancements in nanotheranostics, NSs also integrate diagnostic capabilities, enabling drug tracking, image-guided therapies, and monitoring post-treatment responses, paving the way for highly effective and personalized cancer treatment strategies (Gowda et al., 2024; Gupta et al., 2024a; Ladju et al., 2022).

### 8.1 Breast cancer

**Fig. 5:**
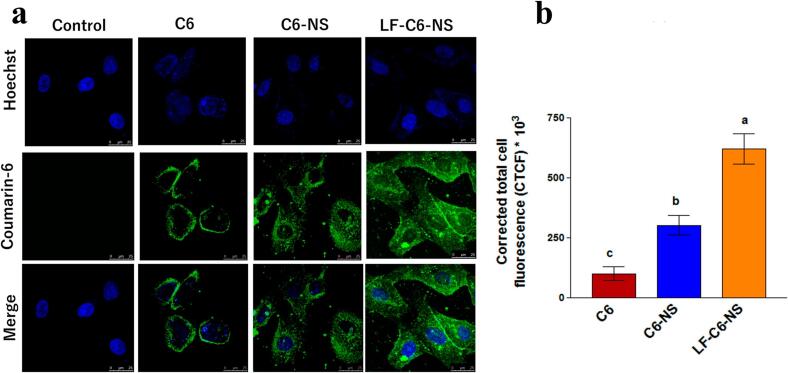
A) Confocal laser scanning microscopy images demonstrate cellular uptake of free coumarin 6 solution and its NS formulations in MDA-MB-231 cells after 4 h incubation, with B) corrected total fluorescence intensity analyzed. Data are presented as means ± SD (*n* = 3) and compared using ANOVA with Tukey's post-hoc test (*p* ≤ 0.05. a > b > c (Aboushanab et al., 2023).

Breast cancer is a leading cause of cancer-related mortality among women, with incidence projected to increase substantially in the coming decades. Early detection improves survival rates, but metastatic forms remain challenging to treat. NSs have emerged as versatile platforms for breast cancer therapy, capable of encapsulating a wide range of therapeutics to enable targeted drug delivery while minimizing toxicity (Argenziano et al., 2020b; Sharma et al., 2022b). The study by Aboushanab et al. fabricated fisetin-loaded CD NSs (FS-NS) coated with lactoferrin (LF) to enhance FS bioavailability and anticancer efficacy for breast cancer. B-CD NSs were optimized using DPC, a cross-linker, achieving high entrapment efficiency (>95 %) and small size (∼ 38 nm). NS showed a mesoporous structure (26 nm pores) with a high surface area and enhanced colloidal stability. LF coating, driven by electrostatic interactions, improved cellular uptake, sustained drug release, and cytotoxicity against MDA-MB-231 cells, reducing IC_50_ by 2.1-fold. Cellular uptake studies of C6-labeled NS and LF-NS showed significantly higher cellular uptake in MDA-MB-231 cells than free dye, with LF-NS demonstrating a 6-fold increase. The enhanced uptake attributed to the small size of the particles and LF interaction with cancer cell receptors contributed to improving antiproliferative and apoptotic effects (Fig. 5, a and b). *In vitro* assays confirmed enhanced apoptosis, migration inhibition, and Fickian diffusion-controlled release (Aboushanab et al., 2023).

Anwer et al. developed a sustained-release NSs of abemacilib (AC) using ethyl cellulose and kolliphor P-188 *via* the emulsion-solvent diffusion method. The optimized AC-NS demonstrated enhanced drug release behaviour, with an initial burst release within 4 h, followed by sustained release over 24 h, achieving 77.12 % drug release compared to 49.33 % for the pure drug. Release kinetics analysis revealed the Higuchi model as the best fit, indicating a diffusion-controlled release mechanism, with a non-Fickian release pattern confirmed by the Korsmeyer-Peppas model. MTT assays showed concentration-dependent cytotoxicity of AC and AC-NS against MCF-7 and MBA-MB-231 cells. The IC_50_ value of AC-NS was 1.2-fold lower than that of pure AC for MCF-7 cells, and for MDA-MB-231 cells, the IC_50_ values of AC and AC-NS were comparable, with a negligible difference (∼1.0-fold). This indicated a slightly enhanced AC-NS in reducing cell viability in MCF-7 cells (Anwer et al., 2022a, Anwer et al., 2022b). Answer MK and colleagues aimed to enhance diosmin (DSM) oral bioavailability, antioxidant properties, and cytotoxic effects of DSM by encapsulating it in β-CD-based NS. The phase solubility and molecular docking studies confirmed strong interactions and inclusion of complex formation between DSM and β-CD, with high solubility constants and hydrogen bonding. The optimized DSM-b-CDNSs demonstrated enhanced drug loading and improved antioxidant activity (94 % at 100 mg/mL). MTT assays revealed that DSM-β-CDNSs significantly reduced the viability of MCF-7 breast cancer cells, with an IC_50_ of 3.8-fold lower than that of free DSM. Enhanced anticancer effects were attributed to improved drug release and increased apoptosis markers (caspase-3, caspase-9, and p53), offering the potential for breast cancer therapy (Anwer et al., 2023). In another study, a hydrogel system was developed for the transdermal co-delivery of curcumin (CUR) and resveratrol (RES) using CD-NSs as a carrier by Pushpalatha et al. CUR-CDNS and RES-CDNS exhibited nanoscale sizes, high drug-loading capacity, and porous structures that facilitate enhanced drug release. CUR-CDNS demonstrated a 10-fold increase in CUR release, while RES-CDNS showed a 2.5-fold improvement compared to free forms. A hydrogel incorporating CUR-CDNS (0.75 % *w*/w) and RES-CDNS (0.25 % w/w) was optimized using Box-Behnken design, achieving maximum flux and semi-stiff consistency. The hydrogel exhibited sustained release kinetics, improved stability, and enhanced transdermal penetration with a 5- and 7-fold increase in the half-life of CUR and RES, respectively. *Ex vivo* studies confirmed significant enhancement in skin penetration due to the nanocarrier system. The cytotoxicity studies on MCF-7 cells revealed a strong synergistic effect at a 3:1 CUR: RES-CDNS ratio, significantly reducing IC_50_ values and supporting enhanced anticancer activity through increased cellular uptake and apoptosis (Pushpalatha et al., 2019).

### 8.2 Lung cancer

**Fig. 6:**
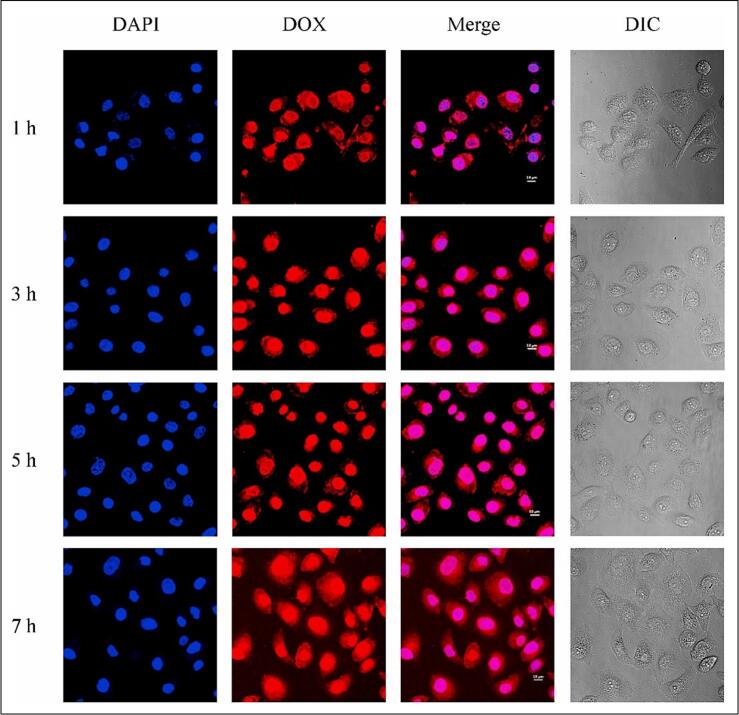
CLSM images of A549 cells incubated with DOX@NS (10 mg DOX equiv./mL) for 1, 3, 5 and 7 h, respectively. Scale bar: 10 μm. Adapted with permission from (Dai et al., 2021).

Lung cancer remains a predominant cause of cancer-related mortality globally, with non-small cell lung cancer (NSCLC) representing approximately 85 % of diagnosed cases. NSs have emerged as a promising therapeutic platform for lung cancer management by improving drug solubility, stability, and targeted delivery while minimizing systemic toxicity. This advanced approach offers significant potential for enhancing therapeutic efficacy in advancing personalized treatment modalities for lung cancer (Koppula and Maddi, 2024). Deinavizadeh et al. synthesized β-CDNS PEG functionalized, encapsulating Au nanorods (AuNRs) and DOX (AuNR-S-PEG.β-CDNS-DOX) for synergistic chemo-photothermal therapy for lung cancer. The AuNR-S-PEG.β-CDNS-DOX was prepared through a multistep reaction. It exhibited efficient photothermal conversion under NIR-laser irradiation, achieving sufficient temperatures for hyperthermal-based cancer therapy. Additionally, the AuNR-S-PEG.β-CDNS-DOX demonstrated pH and laser-responsive drug release, significantly enhancing DOX release under acidic conditions and NIR. In acidic tumor-like conditions (pH 5.5), DOX release increased, achieving 20 % release in 24 h compared to 13 % at pH 7.4. Under 808 nm NIR laser irradiation, the photothermal effect of AuNRs amplified drug release to 64 % at neutral pH and 92 % at pH 5.5. Cellular uptake studies in A549 cancer cells confirmed concentration-dependent internalization. Cytotoxicity studies showed that the AuNR-S-PEG.β-CDNS-DOX was biocompatible at low concentrations and exhibited enhanced anticancer efficacy when loaded with DOX. The photothermal effect significantly reduced cancer cell viability upon NIR irradiation, particularly at higher concentrations. AuNR-S-PEG.β-CDNS-DOX showed enhanced cytotoxicity and synergy between chemotherapy and photothermal therapy with improved cell death efficiency compared to free DOX, making it a promising candidate for targeted cancer treatment (Deinavizadeh et al., 2023). In another study by Almutairy et al., Olmesartan medoxomil (OLM) was fabricated using ethylcellulose (EC)-based NS (OLM-NS) to enhance oral bioavailability and therapeutic efficacy. The NSs demonstrated sustained drug release, achieving 89.5 % release over 24 h compared to 97.36 % of free OLM in 4 h, attributed to the polymer's swelling nature and drug amorphization. *In vitro* cytotoxicity studies against A549 lung cancer cells revealed significant dose-dependent cytotoxicity with OLM-NS showing an IC_50_ of 2.43-fold more than free OLM. Mechanistically, OLM inhibition of RAS and NF-κB pathways contributed to its anticancer activity. In the *in vivo* antihypertensive study, the optimized OLM-NS demonstrated superior efficacy in reducing systolic blood pressure compared to pure OLM, indicating improved antihypertensive efficacy. These results highlighted the ability to prolong drug action, improve bioavailability, and potentially enhance therapeutic outcomes (Almutairy et al., 2021). A novel glutathione (GSH)/pH dual-responsive degradable NS was developed by Dai et al. using β-CD hyper-cross-linked polymer to enhance targeted DOX release for tumor therapy. The DOX-NS demonstrated controlled drug release under acidic and redox TMEs, with a drug release of ∼77 % in pH 5.0 and 10 mM GSH over 96 h, minimizing premature leakage at physiological pH (7.4). Cellular uptake studies confirmed endocytosis-mediated internalization, with intracellular GSH-triggered disintegration releasing DOX into the nucleus (Fig. 6). Antitumor assays against A549 cells revealed an IC_50_ of 1.201 mg/mL of DOX-NS, highlighting the system's efficacy in enhancing drug bioavailability and reducing off-target toxicity (Dai et al., 2021).

### 8.3 Prostate cancer

NSs represent a promising strategy for prostate cancer therapy due to their unique ability to encapsulate a variety of anticancer drugs, shield them from degradation, and ensure targeted delivery to tumor cells. Allahyari et al. fabricate CDNS encapsulated with flutamide (FLT), a prostate cancer drug with low oral bioavailability, to improve solubility and bioavailability. The *in vitro* release studies demonstrated complete FLT release within 3 h from CDNS, with improved drug dissolution attributed to increased wettability due to CDNS hydroxyl groups. The degree of cross-linking significantly influenced drug release rates, with CDNS 1:4 exhibiting faster release than 1:2 due to smaller cavities and higher surface complexity. Cytotoxicity assays on PC3 cells revealed that while blank CDNS were non-toxic, FLT-CDNSs reduced cell viability at higher concentrations through free FLT and exhibited greater immediate cytotoxicity due to faster cell access. Flow cytometry studies confirmed efficient, time-dependent cellular internalization of rhodamine B-loaded CDNSs, particularly for the CDNS 1:4 formulations (Allahyari et al., 2021). In another study, Adrian Matencio and colleagues explored the complexation of oxyresveratrol (OXY) with CDNS and studied the potential in prostate and colon cancer cells. Its characterization confirmed the successful inclusion of OXY into b-CD 1:4 NS. The release profile of OXY provides controlled and prolonged drug release with significantly slower dissolution compared to free OXY. At physiological (pH 7.4) and slightly acidic (pH 5.5) conditions, OXY-CDNS released 45 % and 39 % of the drug in 12 h, while free OXY achieved over 70 % release. Complete release from OXY-CDNS required 80 h, indicating controlled drug delivery. *In vitro* digestion studies demonstrated enhanced stability and bio-accessibility of OXY-CDNS complexes, with sustained release attributed to bile salt displacement in the intestine. Cytotoxicity assays revealed superior dose-dependent antiproliferative effects of OXY-CDNS against PC-3 (prostate cancer), HT-29, and HCT-116 (colon cancer) compared to free OXY, with minimal toxicity from blank NSs. OXY-CDNS showed significantly higher inhibition of cell viability than free OXY, particularly at higher concentrations. Against PC-3 cells viability, OXY-CDNS exhibited 58 % inhibition compared to 43 % for free OXY. HT-29 was more sensitive in colon cancer cells, with 79 % inhibition for OXY-CDNS *versus* 24 % for free OXY, suggesting potential gender-linked effects. The prolonged and controlled release from CDNS likely enhances OXY stability and bioactivity, contributing to its improved antiproliferative efficacy (Matencio et al., 2020). Argenziano et al. encapsulated MEB55 and ST362 analogues of strigolactone (SL) into GSH/pH-responsive NSs for targeted delivery to prostate cancer. The NSs exhibited high encapsulation efficiencies (88.7 % for MEB55, 96.5 % for SR362) and significantly enhanced SL solubility from <0.5 mg/mL to 1.5 mg/mL. The SL-loaded GSH/pH-NS demonstrated redox/pH-responsive release behaviour, with enhanced drug release at elevated GSH levels (2.5-fold higher at 20 mM) and acidic pH 5.5 (3-fold higher than pH 7.4). DU-145 prostate cancer cells, with high intracellular GSH levels, exhibited efficient nanoparticle internalization, as confirmed by flow cytometry and fluorescence imaging, showing greater uptake compared to low-GSH PC-3 cells. SL-loaded GSH/pH-NS showed superior antiproliferative and apoptotic effects compared to free SLs, with minimal toxicity to normal cells, demonstrating their potential as a stimulus-responsive therapeutic platform (Argenziano et al., 2018).

### 8.4 Brain cancer

Brain tumors, including gliomas, which constitute approximately 80 % of cases, are a significant cause of cancer-related mortality worldwide. Current treatments offer limited improvements in survival rates, mainly due to the challenge of delivering anticancer agents across the blood-brain barrier (Kurawattimath et al., 2023; Wu et al., 2021). NSs are emerging as promising nanotechnology-based platforms to address the issue. Fan et al. developed hierarchical self-uncloaking CRISPR-Cas13a RNA nanococoons (RNCOs-D) for targeted cancer therapy, integrating tumor-specific recognition and spatially controlled activation of Cas13a. RNCOs-D compromise RNA NSs (RNSs) for targeted delivery and drug encapsulation and nanocapsules (NCs) that cloak Cas13a ribonucleoprotein (RNP) activity. RNCOs demonstrated tumor-specific targeting, effective lysosomal escape, and robust gene silencing in GBM models. RNCOs-D exhibited superior *in vitro* cytotoxicity against U87-EGFRvIII gene silencing and DOX chemotherapy. Gene silencing achieved ∼70 % protein and ∼ 75 % mRNA downregulation of EGFRvIII, enhancing chemosensitivity and inducing apoptosis *via* caspase activation and Bax/Bcl-2 modulation. *In vivo*, RNCOs-D effectively accumulated at tumor sites, showing a 2.9-fold higher concentration than controls with negligible off-target effects or systemic toxicity. Tumor growth inhibition reached ∼78 %, supported by histopathological analyses confirming apoptosis and reduced proliferation. The findings validate the synergistic therapeutic potential of RNCO-D for GBM (Fan et al., 2022). Peptide-based NS, featuring interwoven hydrophilic and hydrophobic domains, was developed by Yapa et al. to enhance the delivery and efficacy of POH for GBM treatment. Two types, (D-POH)10 K20 and (D-POH)10R20, were evaluated for their ability to deliver POH to glioma cells (GL26) and neural progenitor cells (NPCs). (D-POH)10 K20 demonstrated superior cellular uptake *via* endocytosis and significant cytotoxicity against GL26 cells, even at low concentrations, whereas (D-POH)10R20 showed negligible activity. The cytotoxicity was attributed to enhanced cellular uptake rather than enzymatic cleavage by caspase-6. Notably, POH alone exhibited minimal toxicity due to poor uptake. Similarly, unloaded NSs were non-toxic to both cell types, establishing their biocompatibility. Upon loading with POH, (D-POH)10 K20 NSs showed significant cytotoxicity against GL26 cells after 24 h and 48 h, even in serum-containing media, due to efficient cellular uptake mediated by K20 block. In contrast, (D-POH)10R20 NSs showed minimal toxicity. These NSs provide a promising platform for GBM therapy, with (D-POH)10 K20 showing potential for targeted and effective cancer cell eradication (Yapa et al., 2019).

### 8.5 Colorectal cancer

**Fig. 7:**
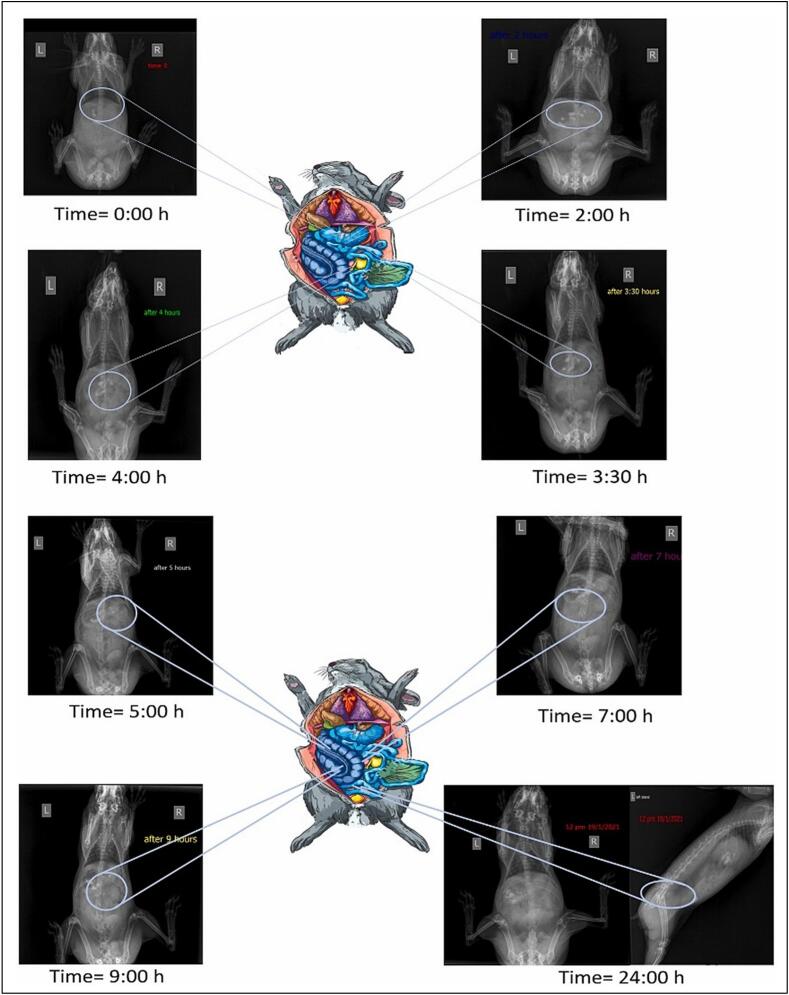
*In vivo* roentgenographic evaluation results of the Coated-MB5 made in fasted white New Zealand healthy rabbits (n = 3, weight: 3–3.5 kg) after receiving the coated microbeads orally; images were taken at 0, 2, 3.3, 4, 5, 7, 9, 24 h after microbeads oral administration. Adapted with permission from (Mashaqbeh et al., 2024).

NSs can encapsulate chemotherapeutic drugs, protecting them from degradation and delivering them in controlled release within the TME. Functionalized with targeting ligands, the NSs can selectively deliver the drug to colorectal cancer cells and minimize systemic toxicity. NSs can be specifically designed to release the drug upon exposure to tumor stimuli such as an acidic environment, over-expressed tumor enzymes, or metabolism specific to the tumor, to obtain precise drug release at the tumor site (Koppula and Maddi, 2024; Mohite et al., 2024). Mashaqbeh et al. developed a colon-specific delivery system utilizing NSs immobilized on polymeric microbeads to co-deliver 5-FU and CUR for colorectal cancer treatment. CUR-NS showed more soluble and amorphous features. The alginate-chitosan microbeads (1:1 weight ratio) were loaded with CUR-NS and 5-FU using optimization that relied on physicochemical parameters, swelling behaviour, and drug release profiles. Coating eudragit and ethylcellulose on the microbeads allowed for sustained and controlled release to ultimately deliver drug specifically to the colon *in vitro* and *in vivo*. The degree of cytotoxicity of the microbeads was measured on HCT116 colorectal cancer cells using an MTT assay. The drug-loaded microbeads showed major cytotoxicity activity, maintaining drug release and restoring and sustaining a reduction in cell viability over 72 h. The coated microbeads assured a gradual release and substantially improved the therapeutic effect. The colon-targeting capability was confirmed using roentgenographic imaging in rabbits, where barium sulfate-loaded coated microbeads were tracked through the gastrointestinal tract. The roentgenographic images confirmed that coated microbeads reached the colon within 7–9 h, demonstrating successful site-specific drug delivery with minimal systemic exposure (Fig. 7) (Mashaqbeh et al., 2024).

A pH-controlled DNA NS delivery system was developed for co-delivering DOX and capecitabine (CAPs) to colorectal cancer cells by Asakiya et al. DNA-NS synthesized *via* rolling circle amplification (RCA) demonstrated high molecular density and selective targeting due to AS1411 aptamer-functionalized templates. By condensing DNA-NS with polyethyleneimine (PEI), they stabilized it against enzymatic degradation and reduced particle size while changing zeta potential. The delivery system achieved high drug-loading efficiency and prolonged drug release at acidic pH (5.5), showing selective drug release in tumor-like environments. The study investigated the anticancer efficacy of DNA-NS/PEI/AS1411 complexes loaded with DOX and CAPs in SW480 cells. Cellular uptake studies confirmed selective targeting of nucleolin-positive SW480 cells *via* AS1411 aptamer, as evidenced by strong fluorescence signals, while minimal binding occurred in LO2 cells. Treated cells showed increased apoptosis, ROS production, and mitochondrial dysfunction. The combination therapy effectively inhibited SW480 cell proliferation, indicating its potential for targeted cancer treatment (Asakiya et al., 2023). Argenziano et al. assessed GSH-responsive CD-NS in 2D and 3D cancer cell cultures, focusing on two formulations (low disulfide content, GSH-NS B, and high disulfide content, GSH-NS D). Both formulations exhibited similar physicochemical properties but distinct cytotoxicity profiles. GSH-NS B showed higher cytotoxicity across colorectal (HCT116, HT-29) and prostate (DU1145, PC-3) cell lines, except DU145, where GSH-NS D was more effective. GSH-NSs induced cell cycle arrest at the G0/G1 phase and modulated CDK and CDKN mRNA expression without significant ROS generation. In 3D spheroid cultures, both formulations exhibited time-dependent cytotoxicity, with notable differences from monolayer responses, highlighting the impact of 3D models on NS efficacy and evaluation. These findings emphasize the utility of 3D cultures as a bridge between traditional 2D models and *in vivo* studies, enhancing the predictive accuracy of nanoparticle toxicity and drug delivery systems (Argenziano et al., 2020a).

NSs represent a highly versatile and effective drug delivery platform across various cancer types. The unique porous structure allows for drug encapsulation, stability, and controlled release, addressing key limitations of conventional anticancer therapies. NSs have been shown to positively influence bioavailability, cellular uptake, and therapeutic window in breast cancer; NSs have also been shown to work in lung, prostate, and brain cancer as well, by allowing for targeted drug delivery, decreased off-target toxicity from drugs, and enhanced bioactivity from drugs. Appropriately responding to NSs with elements that are sensitive to pH or GSH would optimize the potential of NSs to function, especially when targeting tumor-environment specific features. These advancements in NSs technology pave the way for more effective, personalized, and safer cancer treatment strategies. Further relevant studies have been compiled and are summarized in Table 3.

**Table 3:** Overview of NSs for different types of cancer.

Type of cancer	Nanosponge composition and active ingredient	*In vitro*	*In vivo*	Outcome	Reference
Breast cancer	Ethylcellulose-based NSs loaded with Withaferin-A	MCF-7 cells	Adult female albino BALB/c mice	The NS showed sustained release (12*h*), enhanced cytotoxicity, apoptosis induction, and strong *in vivo* antitumor efficacy.	(Shah et al., 2021)
	Lf-coated β-CD NSs loaded with fisetin	MDA-MB-231 cells	Swiss albino mice	Lf-coated β-CD NSs loaded with fisetin showed improved bioavailability and reduced IC_50_ by 2.1-fold, leading to significant tumor inhibition in mice.	(Aboushanab et al., 2023)
	Ethylcellulose/Kolliphor NSs with abemacilib	MCF-7, MDA-MB-231	–	NSs showed enhanced drug release (77 % *vs.* 49 % for free drug) with slight improvement in cytotoxicity.	(Md. K. Anwer et al., 2022)
	β-CDNSs loaded with diosmin	MCF-7 cells	–	Diosmin-loaded β-CDNSs exhibited a 3.8-fold reduction in IC_50_ with increased caspase activation.	(Anwer et al., 2023)
	CDNS hydrogel containing curcumin and resveratrol	MCF-7 cells	–	CDNS-based hydrogel co-delivering curcumin and resveratrol achieved sustained release and synergistic cytotoxicity in breast cancer cells.	(Pushpalatha et al., 2019)
	Hydroxypropyl-β-CD–based carbonate NSs (crosslinked with carbonyldiimidazole) loaded with naringenin	MCF-7 breast cancer cell line and the L929 murine fibroblast cell line	–	The NSs showed higher drug loading, sustained release, enhanced antiproliferative effect against MCF-7 cells, low toxicity to normal cells.	(Peimanfard et al., 2022)
	β-CD-based NSs crosslinked with Epiclon (CDNS8) loaded with CUR	4 T1 cells and normal breast cells (MCF10A)	–	Improved pharmacokinetics, enhanced anticancer activity, selective cytotoxicity, and potential as a promising CUR delivery system.	(Gholibegloo et al., 2019)
	β-CD-based NSs crosslinked with diphenyl carbonate loaded with ferulic acid (FA)	MCF7 and 4 T1 cells	–	Improved solubility, sustained release, and significantly enhanced anticancer activity of FA, making CD-NS a promising delivery system.	(Rezaei et al., 2019)
Breast cancer; cervical cancer	Water-soluble pillar[6]arene (WP6)-based NSs loaded with DOX and mitoxantrone	HeLa, MRC-5, and MCF-7/Adr cells	–	It showed effective host–guest encapsulation and enhanced cytotoxicity.	(Liu et al., 2020)
Lung cancer	Ethylcellulose NSs containing olmesartan	A549 cells	Spontaneous hypertensive rats	Ethylcellulose NSs encapsulating olmesartan improved oral bioavailability and showed dose-dependent cytotoxicity.	(Almutairy et al., 2021)
	Ethylcellulose-polyvinyl alcohol NSs loaded with brigatinib (BGNS)	A549 cells	–	Stable NSs with high entrapment and sustained release; enhanced cytotoxicity and safety.	(Ahmed et al., 2020)
	β-CD hyper-crosslinked NSs with DOX	A549 cells	–	Hyper-crosslinked β-CDNSs provided GSH/pH-responsive DOX release with stronger cytotoxicity.	(Dai et al., 2021)
	GSH-responsive β-CD-pyromellitic dianhydride NSs loaded with erlotinib hydrochloride (ETB)	A549 cells	BALB/c mice	GSH-responsive NS achieved tumor-specific delivery, prolonged release, higher antiproliferative effect, and reduced systemic exposure.	(Momin et al., 2018)
Prostate cancer	Cross-linked β-CD NSs with diphenyl carbonate (EZL-CDNS) loaded with EZL	–	Wistar rats	EZL-CDNS enhanced the solubility, bioavailability, and sustained release of enzalutamide.	(Medarametla and Radha, 2025)
	CD-based NSs loaded with RES and OXY	DU-145 cells	–	The NSs showed improved solubility, stability, antioxidant activity, and anticancer efficacy of RES and OXY.	(Dhakar et al., 2019)
	β-CDNSs with flutamide	PC-3 cells	–	Flutamide-loaded β-CDNSs exhibited faster dissolution and cytotoxicity at higher concentrations.	(Allahyari et al., 2021)
	β-CDNSs loaded with OXY	PC-3, HT-29, HCT-116 cells	–	OXY-loaded β-CDNSs provided improved antiproliferative activity compared to the free drug.	(Matencio et al., 2020)
	GSH/pH-responsive CDNSs containing Strigolactone analogues	DU-145, PC-3 cells	–	GSH/pH-responsive CDNSs carrying strigolactone analogues enhanced solubility, enabled selective uptake, and triggered apoptosis in tumor cells.	(Argenziano et al., 2018)
Brain cancer	RNA nanococoons (RNCOs-D) and DOX + Cas13a RNP	U87-EGFRvIII cells	GBM mice	NA nanococoons integrating Cas13a RNP with DOX (RNCOs-D) achieved 78 % tumor inhibition and significantly improved survival in GBM mouse models.	(Fan et al., 2022)
	Peptide NS loaded with perillyl alcohol	GL26, NPCs cells	–	Enhanced cellular uptake, cytotoxicity, and biocompatibility in GBM cells.	(Yapa et al., 2019)
Colorectal cancer	CDNS-loaded alginate-chitosan microbeads containing 5-FU and curcumin	HCT116 cell	Rabbits	Enabled colon-specific delivery, sustained release, and potent cytotoxicity *in vitro* and *in vivo.*	(Mashaqbeh et al., 2024)
	DNA-NS (AS1411 aptamer, PEI stabilized) containing DOX and Capecitabine	SW480 cells	–	Facilitated selective DOX/capecitabine uptake, enhanced apoptosis, and increased ROS generation.	(Argenziano et al., 2020a)

**NS** – Nanosponges; **CD** – CD; **Lf** – Lactoferrin; **CUR** – Curcumin; **ROS** – Reactive oxygen species; **GSH** – Glutathione; **RES** – Resveratrol; **OXY** – Oxyresveratrol; **DOX** – Doxorubicin; **GBM** – Glioblastoma; **EZL** – Enzalutamide.

## 9 Theranostic applications of NSs

Theranostic NSs are an emerging technology that combines treatment and diagnosis within one platform, allowing targeted therapy and real-time disease monitoring, with particular relevance in cancer care. They are prepared by crosslinking polymers or biomaterials into a porous three-dimensional structure, which can carry therapeutic agents such as anticancer drugs, gene silencers, or immunotherapies, along with diagnostic tools like fluorescent dyes, MRI or CT contrast materials, and radioactive tracers (Gadade and Pekamwar, 2020; Wang et al., 2020).

Shou-Yuan Sung et al., designed a red blood cell (RBC) membrane-coated NS (RBC@NS) carrying graphene quantum dots and docetaxel (GQD-D) for combined drug and energy delivery. The diagnostic and therapeutic potential of Ct-RBC@NS (Cetuximab–Red Blood Cell Membrane NS) was demonstrated using both 3D tumor spheroids and animal models. In spheroid studies, Ct-RBC@NS showed around 8-fold higher fluorescence intensity than non-targeted NSs after 12 h, confirming superior targeting for imaging. In tumor-bearing mice, *in vivo* imaging revealed that targeted NSs produced 60 % stronger tumor-specific signals within 24 h than non-targeted versions. For therapy, combining chemotherapy (docetaxel) with photothermal effects from GQDs and NIR irradiation led to a dramatic reduction in spheroid viability to just 3 %, *versus* over 70 % for chemotherapy alone, highlighting a strong synergistic effect. The RBC membrane coating also prolonged blood circulation, with over 50 % of coated NSs remaining in circulation after 24 h (compared to only 2 % for uncoated ones). Significantly, NIR irradiation enhanced drug accumulation at the tumor site, achieving an 8-fold higher concentration than non-irradiated controls, underscoring the system's promise for precise cancer imaging and treatment. This biocompatible, scalable system shows great promise for precision cancer therapy and diagnosis (Sung et al., 2019).

Elham Gholibegloo et al., formulated a new magnetic theranostic system by attaching CD NSs (CDNS) to magnetite nanoparticles (Fe_3_O_4_) and decorating them with folic acid to enable tumor targeting (Fe_3_O_4_/CDNS-FA). Curcumin, a hydrophobic anticancer compound, was successfully loaded into the CDNS framework, resulting in the Fe_3_O_4_/CDNS-FA@CUR formulation. This study showed that the folic acid-functionalized nanosystem offers diagnostic and therapeutic benefits with strong cancer selectivity. In MRI tests, cancer cells (M109) with high folate receptor expression displayed a dramatic 4.5-fold signal drop, while normal cells (MCF 10 A) showed only a moderate 2.5-fold decrease. The drug-free carrier was highly biocompatible, keeping cell survival above 80 % even at high doses. When loaded with curcumin, the nanosystem killed cancer cells much more effectively than normal cells, lowering their viability to 29.5 % while allowing normal cells to retain 44.8 % viability. Notably, it was much safer than free curcumin, which was highly toxic to both cell types. Overall, this nanosystem shows strong potential as a safe and targeted tool for cancer diagnosis and treatment (Gholibegloo et al., 2019). Table 4 presents an overview of the diagnostic applications of nanosponges in different cancer models.

**Table 4:** Diagnostic Applications of Nanosponges in Cancer Models.

Sr. No.	Drug/Agent	Cancer Type	Animal Model	Diagnostic Application	Diagnostic Outcome/Result	Reference
1	Curcumin	Madison Lung Carcinoma (M109) and Normal Human Mammary Epithelial Cells (MCF 10 A)	–	Targeted Magnetic Resonance Imaging (MRI)	The T2 MR signal intensity for the nanocarrier in M109 cells was approximately 2-fold higher than in MCF 10 A cells, indicating selective cellular uptake.	(Gholibegloo et al., 2019)
2	MPDA-WS_2_@MnO_2_ Nanoplatforms	4 T1 Murine Breast Cancer	4 T1 tumor-bearing mice	Multimodal Imaging (CT, MSOT, MRI)	The nanoplatforms served as effective trimodality contrast agents for CT, MSOT, and tumor microenvironment-responsive T1-weighted MR imaging, allowing for real-time guidance and monitoring.	(Wang et al., 2019)
3	β-CD-Carbon Quantum Dot (β-CD-CQD) Hybrid NSs	HepG2 (Human Liver Cancer)	–	Fluorescence Imaging	The hybrid NSs exhibited strong, bright blue fluorescence with a high quantum yield (38.0 %), demonstrating their potential for tumor imaging.	(Pei et al., 2018)
4	Graphene Quantum Dots (GQDs)	A549 (Human Lung Cancer) and Orthotopic ALTS1C1 Brain Tumor	A549 tumor-bearing BALB/c nude mice and ALTS1C1 tumor-bearing C57B6/L mice	*In vivo* Imaging	The NSs demonstrated enhanced tumor targeting and accumulation, which was visualized using an *in vivo* imaging system (IVIS).	(Sung et al., 2019)
5	Temoporfin	Head and Neck Squamous Cell Carcinoma (FaDu)	FaDu and FaDu/CAF spheroids	Fluorescence Imaging	NSs increased the penetration and created a more uniform distribution of Temoporfin within the tumor spheroids, as observed through fluorescence microscopy.	(Lamy et al., 2023)

## 10 Pharmacokinetics of NSs

NSs have a porous structure and can encapsulate poorly soluble agents, leading to higher oral bioavailability, extended circulation, and sustained plasma concentrations compared to free drugs, significantly improving drug pharmacokinetics by enhancing solubility, stability, and controlled release. Pharmacokinetic studies consistently demonstrate increased C_max_ and AUC, prolonged half-life, and reduced clearance, reflecting slower drug elimination and more efficient systemic exposure. NSs also enable targeted biodistribution, with preferential accumulation in tumor tissues through the EPR effect, while minimizing off-target organ exposure. Collectively, these properties establish NSs as effective carriers for improving drug absorption, prolonging systemic exposure, and enhancing therapeutic efficacy *in vivo* (Gowda et al., 2024; Iravani and Varma, 2022).

Mane et al. investigated the development of β-CD-based ternary NSs (EXE-CDNS-HPMC E5, EF2) for oral delivery of exemestane (EXE) in breast cancer treatment (Mane et al., 2023). *In vivo* pharmacokinetic evaluation using a female Wistar rat model revealed that EF2 markedly improved systemic exposure of EXE compared with the marketed formulation (Aromasin®). Specifically, EF2 achieved a 2.10-fold increase in oral bioavailability, a 1.37-fold increase in maximum plasma concentration (Cmax), and a prolonged elimination half-life due to a reduced elimination rate constant. These improvements indicate enhanced absorption, extended systemic residence time, and prolonged therapeutic effect of EXE when delivered *via* the NSs. The pharmacokinetic benefits translated into pharmacodynamic superiority, as EF2 significantly reduced tumor burden, improved survival rates (100 % survival *versus* ∼80 % in Aromasin®), and minimized hepatotoxicity. Hematological profiling also confirmed improved systemic safety and no evidence of drug-induced anemia.

**Fig. 8:**
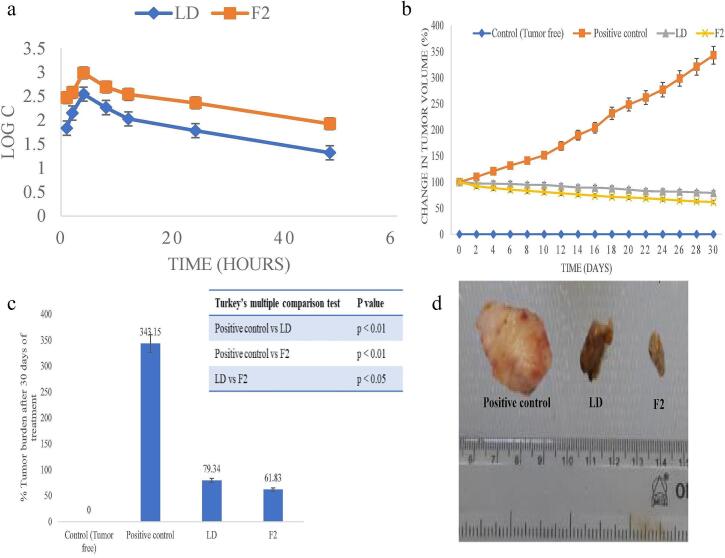
(a) Logarithmic mean plasma concentration–time profiles of LD in rats following a single oral dose (10 mg/kg LD) of the formulated LD-loaded nanosponges (F2) and the pure drug (*n* = 6, ± SD). (b) Time-dependent percentage change in tumor volume in control and treated groups, (c) Percentage tumor burden in control *versus* treated animals (n = 6, *p* < 0.05), (d) Representative digital images of excised breast tumor tissues from experimental animals (Tanaji Mane et al., 2023).

In another study, lapatinib (LD)-loaded ternary β-CD NSs were developed and evaluated for their physicochemical, *in vitro,* and *in vivo* performance to enhance therapeutic efficacy in breast cancer by Tanaji Mane and colleagues (Tanaji Mane et al., 2023). *In vivo* pharmacokinetic investigations in rats demonstrated that formulation F2 significantly improved systemic exposure of LD. The NS-based delivery system increased oral bioavailability by 3.34-fold and maximum plasma concentration (C_max_) by 2.76-fold compared with the pure drug. Additionally, the absorption rate constant (Ka) increased from 0.133/h (pure LD) to 0.168/h (F2), confirming enhanced absorption efficiency. These improvements were directly attributed to the NSs' encapsulation capacity, particle size reduction, and LD transformation into a weakly crystalline state, leading to higher solubility and prolonged systemic circulation. The pharmacodynamic study showed a marked decrease in tumor volume, improved survival (100 % survival with F2 *vs.* ∼34 % mortality with pure LD), favorable hematological parameters, minimal hepatotoxicity, and histopathological features comparable to normal tissue (Fig. 8).

**Fig. 9:**
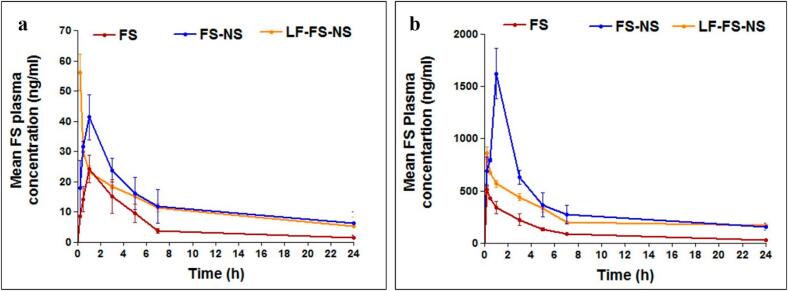
(a) Plasma concentration–time profiles of FS solution and NS formulations after oral administration and (b) intraperitoneal administration of a single 35 mg/kg dose in rats (mean ± SD, n = 6) (Aboushanab et al., 2023).

A study by Aboushanab et al., fisetin (FS)-loaded CD NSs coated with lactoferrin (Lf) to enhance FS oral bioavailability and anticancer effect (Aboushanab et al., 2023). The cytotoxicity, apoptosis, migration, and uptake studies confirmed the superior anticancer potential of FS-loaded NSs. FS-NS reduced the IC_50_ of FS by 1.3-fold, while LF-coated FS-NS further enhanced cytotoxicity with a 2.1-fold decrease in IC_50_ compared to free FS. The pharmacokinetic behaviour of Lf-FS-NS was compared with FS suspension and uncoated FS-NS following both oral and intraperitoneal (IP) administration in female Wistar rats. Upon oral administration, LF-FS-NS achieved a markedly reduced t_max_ (0.25 h) relative to FS suspension and FS-NS (1 h), reflecting faster absorption, likely due to the cationic surface charge of LF-FS-NS that enhances intestinal uptake *via* multiple endocytosis pathways and paracellular transport. Both NS formulations significantly increased systemic exposure: FS-NS enhanced Cmax 1.7-fold and AUC 3.1-fold, while LF-FS-NS increased Cmax 2.3-fold and AUC 2.5-fold compared with FS suspension. Notably, LF-FS-NS exhibited a shorter half-life than FS-NS, consistent with increased clearance of cationic particles *via* serum protein opsonization and macrophage uptake. Following IP administration, both NS formulations demonstrated enhanced bioavailability compared to the FS suspension. FS-NS increased C_max_ and AUC by ∼3-fold and 4.3-fold, respectively, while LF-FS-NS achieved ∼1.7-fold and 3.2-fold increases. As with oral dosing, the half-life of FS was significantly prolonged upon NS loading, but shorter for LF-FS-NS than uncoated FS-NS, suggesting charge-dependent systemic clearance. Additionally, FS bioavailability was consistently higher after IP *versus* oral administration across all formulations, attributable to bypassing intestinal and hepatic first-pass metabolism and the rapid absorption of the drug from the peritoneal cavity. The pharmacokinetic data confirm that NS-based delivery markedly improves FS absorption, systemic exposure, and circulation time compared with FS suspension. While uncoated FS-NS provided the greatest extent of exposure (AUC), LF-FS-NS offered faster absorption and higher peak plasma levels, attributable to its positive surface charge and active tumor-targeting capability (Fig. 9).

A glutathione (GSH)-responsive NS (ETB-NS) was developed for targeted and sustained delivery of erlotinib hydrochloride (ETB) to lung cancer, aiming to mitigate the systemic toxicity associated with conventional ETB administration (Momin et al., 2018). *In vivo* studies in BALB/c nude mice bearing A549 xenografts confirmed significantly enhanced antitumor efficacy of ETB-NS compared with free ETB. Tumor volume was reduced by 97.7 % with ETB-NS *versus* 48.5 % with plain ETB after 28 days. This highlights superior therapeutic performance at lower doses due to controlled intracellular release in the reductive tumor environment. Pharmacokinetic analysis showed prolonged circulation and sustained availability of ETB from NS. Biodistribution studies revealed preferential accumulation of ETB-NS in tumor tissues, with up to ∼15-fold higher levels than in major off-target organs (heart, liver, kidney, spleen). At the same time, negligible drug was detected in the brain, confirming the inability to cross the BBB. This selective localization was attributed to the EPR effect and GSH-mediated release. Collectively, ETB-NS demonstrated controlled pharmacokinetics, tumor-specific biodistribution, and markedly improved *in vivo* antitumor efficacy, underscoring its potential to enhance lung cancer therapy while minimizing systemic toxicity. Table 5 presents a comparative pharmacokinetic analysis of nanosponges with other nanocarrier systems.

**Table 5:** Comparative Pharmacokinetic Analysis between Nanosponges and Other Nanocarriers.

Sr. No.	Drug	Carrier	Dose	Route of administration	Pharmacokinetic Parameters	Comparative Advantages of NSs
1	Fisetin	Liposome (Seguin et al., 2013)	13 mg/kg	IV	Cmax: 10,000 ng/mL T-half: 3.8 h AUC: 1840 ng.h/mL	Superior Sustained Delivery & Bioavailability. Provides a 3.2-fold longer T-half and doubles the total drug exposure (AUC), making it far more effective for sustained therapy.
		NSs (Aboushanab et al., 2023)	30 mg/kg	Oral/IP	Cmax: 1622.4 ng/mL Tmax: 1 h T-half: 17.7 h AUC: 8474.5 ng.h/mL Bioavailability: 4.3 % (relative)	
2	Paclitaxel	NLC (Yang et al., 2013)	3 mg/kg	IV	Cmax: 2830 ng/mL Tmax: 0.083 h T-half: 2.17 h AUC: 5210 ng.h/mL	Vastly Superior Drug Exposure. Achieves a 7.5-fold higher Cmax and an 11-fold greater total drug exposure (AUC). It is unequivocally the more potent delivery system for this drug.
		NSs (Torne et al., 2010)	10 mg/kg	Oral	Cmax: 21,239 ng/mL AUC: 24,128,400 ng.h/mL Bioavailability: 256 % (relative)	
3	Tamoxifen	SNEDDS (Shrivastava et al., 2022)	1.02 mg/kg	Oral	Cmax: 321.48 ng/mL Tmax: 3 h AUC: 3737.3 ng.h/mL	Faster & More Potent Onset. Delivers a 31 % higher peak concentration (Cmax) and achieves it almost 3 times faster (Tmax), indicating a more rapid and powerful therapeutic effect.
		NSs (Torne et al., 2013)	4 mg/kg	Oral	Cmax: 421,156 ng/mL Tmax: 1.0179 h T-half: 1.018 h AUC: 1,171,630 ng.h/mL Bioavailability: 145 % (relative)	
4	Entrectinib	Nanosuspension (Chary et al., 2024)	60 mg/kg	Oral	Cmax: 5226.54 ng/mL Tmax: 2 h T-half: 1.24 h AUC: 14,958.9 ng.h/mL	Superior Across Key Metrics. Achieves a 58 % higher Cmax, a 4.9-fold longer T-half, and a 3.6-fold greater total drug exposure (AUC), demonstrating overwhelming superiority.
		NSs (Reddy and Bhikshapathi, 2024)	30 mg/kg	Oral	Cmax: 8290.66 ng/mL Tmax: 3 h T-half: 6.079 h AUC: 53,587.3 ng.h/mL	
5	Exemestane	Liposome (Sharma et al., 2025)	15 mg/kg	Oral	Cmax: 234,100 ng/mL Tmax: 4 h T-half: 9.37 h AUC: 3071.03 ng.h/mL	Superior Sustained Action & Faster Onset. Provides a 3-fold longer half-life, a 1.7-fold greater total drug exposure (AUC), and reaches peak concentration twice as fast.
		NSs (Mane et al., 2023)	30 mg/kg	Oral	Cmax: 170.19 ng/mL Tmax: 2 h T-half: 27.94 h AUC: 5135.7 ng.h/mL	
6	Lapatinib	NPs (Wan et al., 2015)	10 mg/kg	IV	Cmax: 19,434 ng/mL Tmax: 0 h T-half: 6.19 h AUC: 113,718.2 ng.h/mL Bioavailability: 100 % (absolute)	Significantly Better for Sustained Oral Delivery. NSs show a 3-fold longer T-half (18.57 h *vs* 6.19 h), ideal for sustained release. While the IV nanoparticles have a higher AUC, the oral NSs achieve a 3.34-fold bioavailability enhancement over the pure oral drug, proving their effectiveness for oral administration.
		NSs (Tanaji Mane et al., 2023)	10 mg/kg	Oral	Cmax: 971.08 ng/mL Tmax: 4 h T-half: 18.57 h AUC: 15,792.1 ng.h/mL Bioavailability: 334 % (relative)	

Taken together, NS-based drug delivery systems enhance pharmacokinetic performance by improving solubility, absorption, and systemic exposure while enabling sustained release and targeted biodistribution. Across multiple studies, NSs consistently increased oral bioavailability (2–6-fold), elevated C_max_, prolonged half-life, and reduced clearance compared to free drugs. These improvements translated into superior pharmacodynamic outcomes, including enhanced tumor regression, prolonged survival, and reduced systemic toxicity.

## 11 Toxicity aspects

To ensure that different NSs are safe for use, their toxicological properties must be assessed at the cellular level and then at the preclinical and clinical levels. Numerous dosages and acute oral and topical toxicity experiments have been conducted on carbonate and pyromellitic NSs, and the results have demonstrated high tolerance. β-CD-NSs were initially assessed for the lack of cytotoxicity following 24 and 48 h experiments on a variety of cell lines, including HaCaT, HEK(a), HELA, MCF7, COS, Vero, HT-29, and HCPC-1, as well as the lack of hemolytic activity on erythrocytes up to a concentration of 20 mg/mL (Mullick et al., 2022). Additionally, topical application assessed them for both acute and chronic toxicity in Swiss Albino and Balb/c mice, with no adverse effects seen (Rubin Pedrazzo et al., 2019). In a separate study, Shende P et al. assessed the acute and repeated dosage toxicity of several NS formulations (S1–S6) synthesized using various cross-linking techniques per OECD rules 423 and 407, respectively. To assess acute toxicity, all formulations were given to female rats. The rats were observed for 14 days before being killed and having a necropsy performed. Rats of both sexes were given formulations at a dosage for 28 days to conduct a repeated dose toxicity study. The experimental animals' hematological and biochemical parameters did not significantly alter after 28 days of repeated formulation administration. These findings show that when tested on experimental animals, the formulations are safe (Singh et al., 2024).

## 12 Clinical translation and patent

### 12.1 Large-scale synthesis

Scaling up the synthesis of NSs from laboratory to industrial scales presents complex challenges, but also offers significant opportunities for industrial manufacturing, particularly in the field of anticancer therapies. The inherent complexity of chemical crosslinking and NS fabrication necessitates meticulous control over reaction parameters. Addressing these challenges will lead to advancements in manufacturing processes (Vasquez, 2024). For instance, the resolution of scale-up challenges has led to the emergence of novel, environmentally sustainable, and efficient synthesis methodologies, such as microwave and ultrasound-assisted polymerization. These techniques enhance reaction kinetics and improve the morphological uniformity of NS structures. Such technological advancements not only augment production efficiency but also facilitate the attainment of consistent product quality, which is paramount for clinical applicability and scale-up synthesis (Aneja et al., 2025). Moreover, the imperative for reliable batch consistency has prompted the integration of real-time monitoring capabilities and automated process controls. This integration ensures that each batch of NSs achieves reproducible drug loading and release profiles critical determinants of therapeutic efficacy and regulatory compliance (Sharma, 2024). The complexities associated with scale-up have further stimulated the optimization of reaction conditions, purification processes, and drying methodologies specifically tailored for large volume production (Majee and Biswas, n.d). These optimizations yield a more cost-effective, scalable, and environmentally responsible manufacturing methodology to produce a desirable NS formulation.

### 12.2 Regulatory approval and safety issues

The successful clinical translation of NSs depends on addressing significant regulatory and safety challenges. One of the primary hurdles is navigating the stringent approval processes required for novel nanotechnology-based therapeutics. Regulatory agencies such as the Indian Pharmaceutical Association (IPA), the U.S. Food and Drug Administration (FDA), and the European Medicines Agency (EMA) mandate extensive preclinical evaluations to ensure the safety, efficacy, and quality of NS formulations (Koppula and Maddi, 2024). These assessments must demonstrate that NSs do not cause adverse biological effects such as toxicity, inflammation, or immune reactions, an especially complex task given their nanoscale size and intricate interactions within biological systems.

Safety concerns associated with non-biodegradable NSs primarily arise from their potential to accumulate within the body and the toxic effects of their degradation products if they fail to break down properly. Such NSs, or those that degrade into harmful substances, may persist in tissues or organs and cause chronic inflammation or long-term toxicity (Missaoui et al., 2018). In contrast, NSs fabricated from biodegradable materials are generally regarded as safer, as they tend to exhibit low toxicity and their degradation products are typically non-toxic and easily eliminated from the body. CD-based NSs, for instance, have demonstrated excellent biocompatibility, good clearance, and minimal toxicity in animal studies (Patil et al., 2025, Stanislawska et al., 2019). To address the remaining safety issues, current research focuses on designing fully biodegradable NSs that decompose into harmless molecules and are efficiently metabolized by organs such as the liver. Additionally, surface modification approaches, including coating NSs with biocompatible hydrogels, are being explored to enhance their circulation time while minimizing potential toxic effects (Umut, 2013). To realize their full potential, it is essential to adopt a balanced approach that integrates regulatory compliance, rigorous safety assessment, and scalable production strategies (Koppula and Maddi, 2024; Sufian et al., 2017). Overcoming these challenges will be key to translating NS-based systems from experimental research to safe and effective clinical use.

### 12.3 Cost effectiveness

The cost-effectiveness issue is one of the major reasons that hinder the use of nanoparticle systems (NSs) in cancer treatment, although their therapeutic capabilities are great. The NSs provide benefits such as targeted drug delivery, reduced toxicity, and more potent treatment; however, the expenses associated with their research, large production, and clinical use are still a big issue. The process of fabricating the NSs typically requires polymers, crosslinkers, and cutting-edge technology, for example, emulsion solvent diffusion, ultrasound-assisted, and microwave-assisted synthesis methods. These fabrication methods can result in increased production costs compared to traditional drug formulations. With NSs, there are also additional costs related to quality control, purification, and batch-to-batch consistency, which add further costs to the overall process, especially when scaling up to clinical use is considered. Although NSs will reduce the overall amount of drug required, minimize side effects, and therefore reduce patient treatment costs in the long term, still a large investment into process optimization, equipment, and regulatory compliance is still very high. Moreover, there is still scope in developing a cost effective, green method for large scale production. Although NSs show promise as a cost effective method to implement due to their multifunctionality and potential to improve patient outcomes in the future, large scale synthesis of NSs can be problematic when these costs prohibit clinical application on a widespread basis for cancer therapy (Teja and Radha, 2023). The clinical translation of nanosponges holds considerable promise, yet its success will depend on addressing challenges related to large-scale, reproducible synthesis, regulatory compliance, and cost-effective manufacturing. Continued efforts towards developing sustainable production strategies, improving biocompatibility and biodegradability, and meeting stringent safety requirements are critical for advancing NSs from preclinical studies to clinical practice. By integrating innovative fabrication methods with rigorous regulatory frameworks and economic feasibility, nanosponges can emerge as a transformative platform in precision medicine, particularly for cancer and infectious disease therapies.

### 12.4 Patents

The list of different patents filed on NSs to treat cancer is shown in Table 6.

**Table 6:** List of different patents filed on NSs for cancer.

Patent office	Application Number	Title	Applicant	Publication Date
Republic of Korea	1,020,210,028,401	Temperature-Sensitive Nanosponge Platform For Hydrophobic Drug Delivery And Uses Thereof	Korea Institute Of Ceramic Engineering And Technology	2022-09-14
India	202,321,076,376	Natural Polymer Based Nanosponges Encapsulated Phytochemicals From *Annona Squamosa* L. And Capparis Zeylanica L. For Cancer Treatment	Mrs. Shailaja Amol dombe Dr. Pramodkumar Jaykumar Shirote	2023-12-15

## 13 Conclusion and future prospective

NSs have emerged as a versatile and promising platform for drug delivery, particularly in cancer therapy. They can encapsulate hydrophilic and lipophilic drugs, enhance bioavailability, and provide controlled drug release. Thus, they would be candidates for improving therapeutic efficacy with reduced systemic toxicity. Moreover, their surface functionalization with targeting ligands, such as folic acid, makes selective drug delivery possible, further enhancing their clinical utility. Despite these advancements, the clinical translation of NS-based systems faces several challenges, including synthesis variability, scalability, and regulatory compliance. Furthermore, understanding their biodistribution, degradation kinetics, and interaction with biological environments remains essential for ensuring their safety and efficacy in human applications. Studies should consider refining the physicochemical properties for the further optimization of NSs while also exploring the newly developed methods of fabrication and combining them with the imaging modalities for theranostic applications. Incorporating stimuli-responsive materials will enable real-time monitoring and on-demand drug release, potentially revolutionizing personalized medicine. By addressing the existing challenges and leveraging recent technological advancements, NSs have the potential to play a transformative role in modern medicine. Future NS-based therapies could transition from the laboratory to clinical practice and improve cancer management and beyond, owing to the continuing interdisciplinary collaboration among materials scientists, pharmacologists, and clinicians. Future research on NSs should focus on improving functionalization to reduce toxicity, enhance biosafety, and increase specificity for targeted drug delivery. Modifying their physicochemical properties, such as the concentration of polymer and cross-linkers, can lead to multifunctional NSs with a wide range of applications. Key areas for further investigation include the biodistribution, biocompatibility, and long-term biosafety of NSs. Moreover, their complexion capabilities should be optimized, with structural modification and scalability towards large-scale production should be prioritized. Surface functionalization techniques, such as incorporating fluorescent compounds, magnetite nanoparticles, or targeting ligands like folic acid, could improve the therapeutic potential of NSs for cancer theranostics. The versatility of NS as drug carriers can be incorporated into various dosage forms. Novel dosage forms such as microneedles (MNs), implants, or suppositories may offer localized cancer therapy, though challenges like the low drug loading capacity for lipophilic compounds need to be addressed. Incorporating NSs could enhance the solubility of anticancer drugs in MNs, overcoming their limitations. Despite their potential, NSs face challenges in clinical translation, including controlling their size, porosity, and drug release profiles. Variations in methods of fabrication could affect consistency, making standardization crucial. Comprehensive studies on biocompatibility, toxicity, and *in vivo* behaviour are needed. NSs show great promise in personalized medicine and cancer therapy. Incorporating imaging modalities and similar responsive materials would allow NSs to facilitate real-time monitoring and on-demand drug release, further enhancing therapeutic efficacy. Overcoming scalability, reproducibility, and regularity challenges will be crucial in fully exploiting their potential in medical applications.

## CRediT authorship contribution statement

**Sandesh Ramchandra Jadhav:** Writing – review & editing, Writing – original draft, Methodology, Conceptualization. **Ashutosh Gupta:** Writing – review & editing, Writing – original draft, Methodology, Conceptualization. **Viola Colaco:** Writing – original draft, Methodology. **Moumita Saha:** Writing – original draft, Methodology. **Amatha Sreedevi:** Writing – original draft, Methodology. **Deepanjan Datta:** Writing – review & editing. **Sudheer Moorkoth:** Writing – review & editing, Supervision. **Virendra S. Ligade:** Writing – review & editing. **Namdev Dhas:** Writing – review & editing, Writing – original draft, Validation, Supervision, Methodology, Conceptualization.

## Declaration of competing interest

The authors declare no conflict of interest.

## Data Availability

Data will be made available on request.
